# Ecological restoration enhances dryland carbon stock by reducing surface soil carbon loss due to wind erosion

**DOI:** 10.1073/pnas.2416281121

**Published:** 2024-11-08

**Authors:** Jian Song, Shiqiang Wan, Kesheng Zhang, Songbai Hong, Jianyang Xia, Shilong Piao, Ying-Ping Wang, Jiquan Chen, Dafeng Hui, Yiqi Luo, Shuli Niu, Jingyi Ru, Hao Xu, Mengmei Zheng, Weixing Liu, Haidao Wang, Menghao Tan, Zhenxing Zhou, Jiayin Feng, Xueli Qiu

**Affiliations:** ^a^School of Life Sciences/Hebei Basic Science Center for Biotic Interaction, Institute of Life Science and Green Development, Hebei University, Baoding, Hebei 071002, China; ^b^Luoyang Institute of Science and Technology, Luoyang, Henan 471023, China; ^c^School of Urban Planning and Design, Shenzhen Graduate School, Peking University, Shenzhen 518055, China; ^d^Zhejiang Tiantong Forest Ecosystem National Observation and Research Station, State Key Laboratory of Estuarine and Coastal Research, School of Ecological and Environmental Sciences, East China Normal University, Shanghai 200241, China; ^e^Research Center for Global Change and Complex Ecosystems, Institute of Eco-Chongming, East China Normal University, Shanghai 200241, China; ^f^Institute of Carbon Neutrality, Sino-French Institute for Earth System Science, College of Urban and Environmental Sciences, Peking University, Beijing 100871, China; ^g^State Key Laboratory of Tibetan Plateau Earth System, Resources and Environment, Institute of Tibetan Plateau Research, Chinese Academy of Science, Beijing 100085, China; ^h^Commonwealth Scientific and Industrial Research Organisation (CSIRO) Environment, Clayton South, VIC 3169, Australia; ^i^Center for Global Change and Earth Observations, Department of Geography, Environment and Spatial Sciences, Michigan State University, East Lansing, MI 48823; ^j^Department of Biological Sciences, Tennessee State University, Nashville, TN 37209; ^k^School of Integrative Plant Science, College of Agriculture and Life Sciences, Cornell University, Ithaca, NY 14850; ^l^Key Laboratory of Ecosystem Network Observation and Modeling, Institute of Geographic Sciences and Natural Resources Research, Chinese Academy of Sciences, Beijing 100085, China; ^m^College of Life Sciences, Henan Normal University, Xinxiang, Henan 453007, China; ^n^State Key Laboratory of Efficient Utilization of Arid and Semi-arid Arable Land in Northern China, Institute of Agricultural Resources and Regional Planning, Chinese Academy of Agricultural Sciences, Beijing 10081, China

**Keywords:** carbon neutrality, climate solutions, plant productivity, soil carbon, soil erosion

## Abstract

Enhancing terrestrial carbon (C) stock through ecological restoration, one of the prominent approaches for natural climate solutions, is conventionally considered to be achieved through an ecological pathway, i.e., increased plant C uptake. By conducting a comprehensive regional survey of 4279 1 × 1 m^2^ plots at 517 sites across China’s drylands and a 13-y manipulative experiment in a semiarid grassland within the same region, we show that greater soil and ecosystem C stocks in restored than degraded lands result predominantly from decreased surface soil C loss via suppressed wind erosion. This biophysical pathway is always overlooked in model evaluation of land-based C mitigation strategies. Surprisingly, stimulated plant growth plays a minor role in regulating C stocks under ecological restoration. In addition, the overall enhancement of C stocks in the restored lands increases with both initial degradation intensity and restoration duration. At the national scale, the rate of soil C accumulation (7.87 Tg C y^−1^) due to reduced wind erosion and surface soil C loss under dryland restoration is equal to 38.8% of afforestation and 56.2% of forest protection in China. Incorporating this unique but largely missed biophysical C-conserving mechanism into land surface models will greatly improve global assessments of the potential of land restoration for mitigating climate change.

## Significance

Ecological restoration is widely hypothesized to enhance ecosystem carbon stocks by stimulating plant photosynthetic carbon fixation. However, the role of vegetation in mitigating dryland soil carbon loss from wind erosion is often overlooked. This study distinguishes the contributions of increased plant carbon input and reduced wind erosion carbon loss to ecosystem carbon stocks under ecological restoration through a comprehensive regional survey covering an area of 3.14 million km^2^ and a 13-y manipulative experiment in China’s drylands. Contrary to widely accepted consensus, enhanced plant growth contributes little to increased carbon stocks under ecological restoration, which is predominantly caused by reduced surface soil carbon loss due to suppressed wind erosion. This finding reveals a unique but often-overlooked mechanism enhancing dryland carbon stocks.

The terrestrial biosphere can sequester nearly one-third of anthropogenic CO_2_ emissions (1). The increase in the terrestrial carbon (C) sink, which is considered as one of the predominant natural climate solutions (2–4), is primarily attributed to the photosynthetic capture of C by forests (5, 6). Afforestation has tremendous potential in mitigating the rise in atmospheric CO_2_ concentration and climate change (6–9). For example, the Forest and Land Use Declaration, announced at the COP26 (10), and the latest IPCC special report (11) suggest that expanding forests by 1 billion hectares can be a crucial step toward achieving the 1.5° C climate warming target of the Paris Agreement by 2050 (12). In contrast, drylands are often overlooked in global C stock inventories despite their covering 45% of the Earth’s land surface (13), largely due to the limited potential of C accumulation through afforestation in these vast water-limited regions (14). However, dryland ecosystems have been unambiguously demonstrated to account for 42% of net primary productivity (15) and 65% of increments of gross primary productivity globally from 1982 to 2015 (16), thus playing a dominant role in determining the interannual variability of land CO_2_ sink (17).

Drylands are vulnerable to both natural and anthropogenic perturbations (13, 18, 19). Land degradation over the past decades or centuries, resulting mainly from intensified human activities (20), has led to widespread wind erosion and frequent dust storms in drylands across the world (21–23). Given that 44% of organic matter in the top 30 cm of soil worldwide is stored in dryland ecosystems (24), wind erosion can cause enormous losses of surface soil and associated C and nutrients (25, 26), thereby negatively impacting dryland C stocks (27–29). The UN’s Convention to Combat Desertification recommends implementing ecological restoration to reestablish vegetation patterns and ecosystem functions in degraded drylands, with a particular focus on preserving and enhancing C stocks. Ecological restoration is crucial for the sustainability of the degraded hotspots worldwide, particularly in dryland regions (24, 30). On the one hand, ecological restoration is widely accepted to have the potential to stimulate plant growth and productivity (31, 32), which contributes to regional greening (23, 33) and C sequestration (9, 34, 35). On the other hand, enhanced vegetation cover can in turn strengthen soil surface resistance by decreasing both wind speed and bare soil fractions (36–38), subsequently suppressing dust emissions and associated soil C and nutrient losses in drylands (39–41). Nonetheless, the C cycling schemes in land surface models often overlook the dynamic nature of surface soil and the resulting C losses caused by wind erosion (42). The issue is further exacerbated by the lack of field-based in situ evidence on whether and to what extent reduced C and nutrient losses under ecological restoration regulate plant growth and ecosystem C stocks. An integrated assessment of the relative contributions of ecological (increasing plant C input) and biophysical (reducing wind erosion C loss) pathways to ecosystem C stocks is fundamentally important for projection of the potential of restoring degraded drylands to mitigate climate warming.

## A Comprehensive Regional Survey

**Fig. 1.**
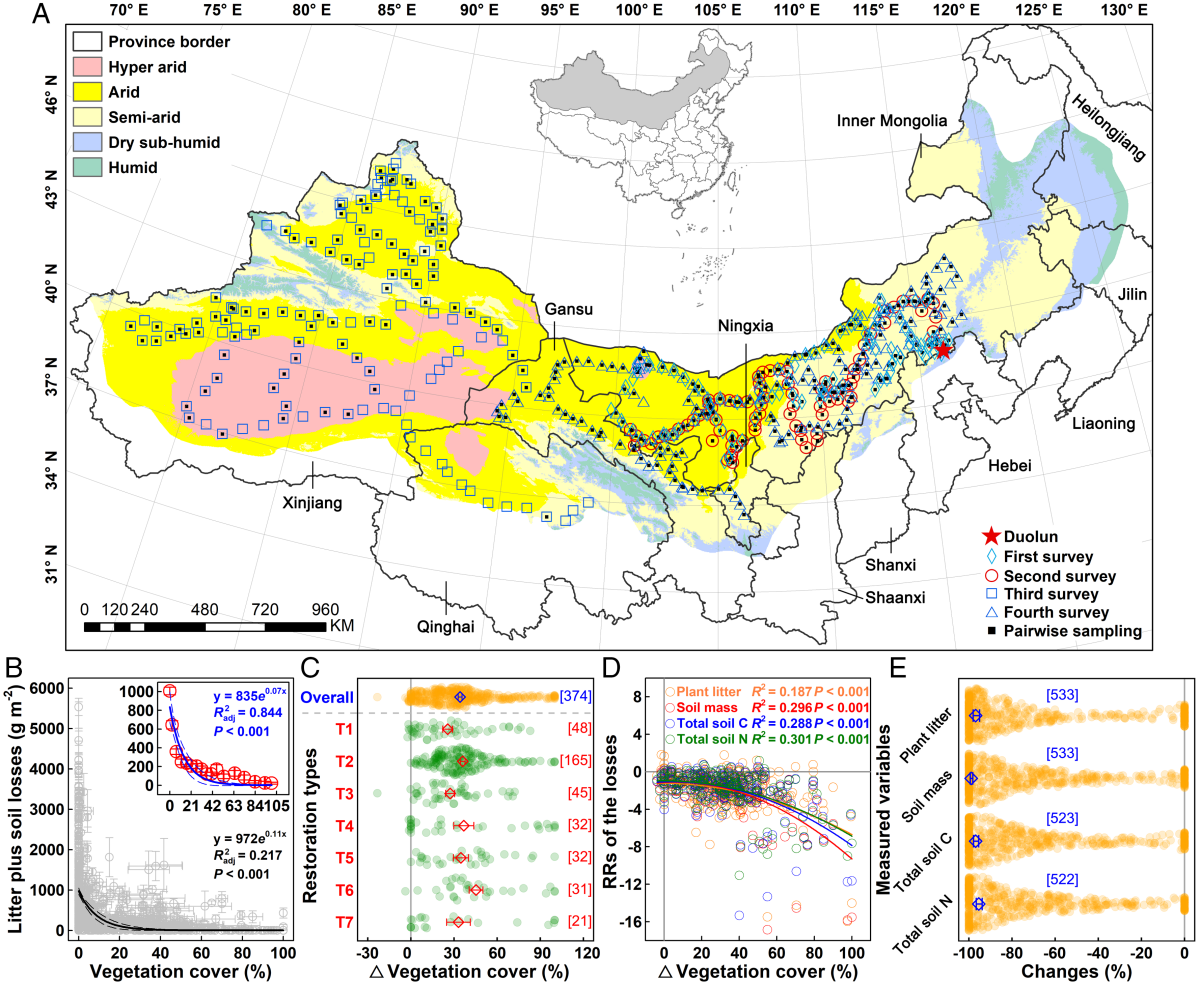
Ecological restoration stimulates vegetation cover and reduces wind erosion across China’s drylands. The locations of sampling sites in the four transect surveys in North and Northwest China and the field experimental site in Duolun County (*A*). Symbols with black solid squares inside indicate the sites where pairwise sampling was used. Spatial dependencies of the wind erosion-induced plant litter and soil dry mass losses on vegetation cover, with the inset showing the relationships using cover data binned at ~6% intervals (*B*). Each gray data point (± 1 SE) represents the mean value of all degraded or restored plots of a single site. Absolute changes in vegetation cover (mean ± 1 SE, diamond with error bar) averaged across all (Overall) and in each of the seven ecological restoration types (T1−T7; *C*). The values in square brackets along the y-axis show sample size (number of sites). Dependencies of response ratios (RRs) of the wind erosion-induced losses of plant litter, soil dry mass, and total soil carbon (*C*) and nitrogen (N) on the changes in vegetation cover under ecological restoration (*D*). Percentage changes (mean ± 95% CI, diamond with error bar) in wind erosion-induced losses of plant litter, soil dry mass, and total soil C and N averaged across all plot pairs (*E*). There is significant change if the 95% CI does not overlap zero. The blue values in square brackets indicate the number of sites (a site is counted multiple times if the site includes wind erosion simulations at different wind speeds). Colored scatter plots in *Panels C*−*E* represent changes/RRs in these variables under 25 m s^−1^ wind erosion (*C* and *D*) and all five wind speeds (*E*) at each site. T1: grazed/degraded vs. ungrazed/undisturbed grasslands, T2: bare lands vs. disturbed/undisturbed grasslands/shrublands, T3: poorly developed vs. well-developed grasslands/shrublands, T4: deserts without vs. with desertification control, T5: croplands vs. natural grasslands, T6: croplands vs. old-field grasslands, and T7: bare soil vs. crop/no tillage with residue retention.

To address these knowledge gaps, we performed a three-year (2014–2016) regional survey with 4279 1 × 1 m^2^ plots across 517 sites, including 1599 plot pairs (degraded vs. restored) at 391 sites, in five provinces of North and Northwest China (Fig. 1*A*, and *Methods* and *SI Appendix*, Table S1). This extensive survey covered an area of 3,142,951 km^2^ and included all semiarid, arid, and hyperarid regions in China, which represent one of the global dryland hotspots (13) and major source regions of dryland wind erosion due to high wind speeds and low precipitation (*SI Appendix*, Fig. S1 and Table S2; ref. 43). Three-North Shelter Forest Program, one of the national ecological restoration programs in China, covers an area of 320,000 km^2^ and serves as one of the critical dust barriers in North China by planting trees. Therefore, our survey regions include, but are much larger than, the regions of Three-North Shelter Forest Program. We first measured vegetation cover and then mimicked wind erosion and collected plant litter and soil lost from each of the sampled plots. The effects of ecological restoration on the measured variables were evaluated using a pairwise sampling scheme that directly compared the differences between adjacent restored and degraded plots in each plot pair (restored minus degraded) at each site (*SI Appendix*, Fig. S2). This approach minimizes the confounding impacts of spatial heterogeneity on the restoration effects at each site and across vast dryland regions.

Because wind speed is a major biophysical factor that affects wind erosion intensity (38, 44, 45), we simulated wind erosion using five wind speeds (12, 16, 21, 25, and 30 m s^−1^) during the first transect survey in spring 2014. Despite the observed increases in plant litter and soil dry mass losses with wind speed (*SI Appendix*, Fig. S3), the restoration effects on plant litter, soil dry mass, and total soil C and nitrogen (N) losses did not differ among the five wind speeds across the 44 sites using pairwise sampling (*SI Appendix*, Fig. S4) or vary with ambient wind speeds either (*SI Appendix*, Fig. S5). This consistency could be explained by similar soil texture, cohesion, and surface resistance in the adjacent degraded and restored plots in each plot pair with distance less than 10 m at each site (*SI Appendix*, Fig. S2). Therefore, a wind speed of 25 m s^−1^, which corresponds to the saturation wind speed to cause maximum plant litter and soil dry mass losses (*SI Appendix*, Fig. S3), was used in the subsequent three transect surveys. Land surface roughness, which is closely related to the fraction of vegetation cover, is another major biophysical factor influencing wind erosion intensity (44, 45), with wind speed at the soil surface generally declining with increasing roughness (28, 36, 38). Data from a specific test sampling in the first transect survey showed that wind speed at the soil surface was significantly reduced in the plots with shrubs comparing to those with bare soil (*SI Appendix*, Fig. S6). Consequently, the total losses of plant litter and soil dry mass due to wind erosion declined exponentially with increasing vegetation cover across all the sampled plots (Fig. 1*B*; *R*^2^ = 0.217, *P* < 0.001), with grouped vegetation cover at 6.0% intervals explaining 84.4% variations in the total losses of plant litter and soil dry mass (Fig. 1*B*, *Inset*). These observations are in line with those of a field monitoring on the Colorado Plateau that modeled aeolian sediment flux declines exponentially with perennial vegetation cover (40).

In drylands, ecological restoration can enhance vegetation cover and surface roughness. This, in turn, decreases both the relative area vulnerable to wind erosion and wind speeds at the soil surface (22, 36, 39–41, 45). Across all seven ecological restoration types, vegetation cover was 34.2 ± 1.4% (absolute change, *P* < 0.001, paired *t* test) greater in the restored than degraded plots (Fig. 1*C*), although the increase magnitudes (all *P* < 0.001) varied with restoration type (*Methods* and *SI Appendix*, Table S3). This result is consistent with the widely accepted consensus that ecological restoration promotes vegetation cover (23, 39). Subsequently, the reduction magnitudes of wind erosion-induced losses of all plant litter (*R*^2^ = 0.187, *P* < 0.001), soil dry mass (*R*^2^ = 0.296, *P* < 0.001), total soil C (*R*^2^ = 0.288, *P* < 0.001), and total soil N (*R*^2^ = 0.301, *P* < 0.001) declined with the increments of vegetation cover under ecological restoration (Fig. 1*D*). Importantly, results of random forest analyses revealed that among the variables including vegetation (cover and height), soil properties (soil moisture and bulk density), local climate conditions (mean annual precipitation and temperature), and geographical positions (latitude, longitude, and elevation), the changes in vegetation cover consistently emerged as the predominant factor in controlling the spatial patterns of the losses of plant litter, soil dry mass, total soil C, and total soil N under wind erosion (*SI Appendix*, Fig. S7). As a result, ecological restoration significantly reduced the losses of all plant litter (mean −96.9%; 95% CI: −95.0%, −98.0%), soil dry mass (−98.9%; −98.2%, −99.4%), total soil C (−96.9%; −95.5%, −97.9%), and total soil N (−95.4%; −93.8%, −96.6%; Fig. 1*E*) averaged across all the plot pairs. Irrespective of the significant variations in the reduction magnitudes among the three ecosystem types (i.e., grasslands, deserts, and croplands prior to ecological restoration) and the seven restoration types (*SI Appendix*, Fig. S8), the almost complete diminishing of soil C and N losses suggest that ecological restoration is a highly effective strategy in ameliorating the negative impacts of wind erosion on ecosystem C and nutrient contents in drylands (23, 46). It acts as a biophysical pathway to preserve dryland C stocks.

**Fig. 2.**
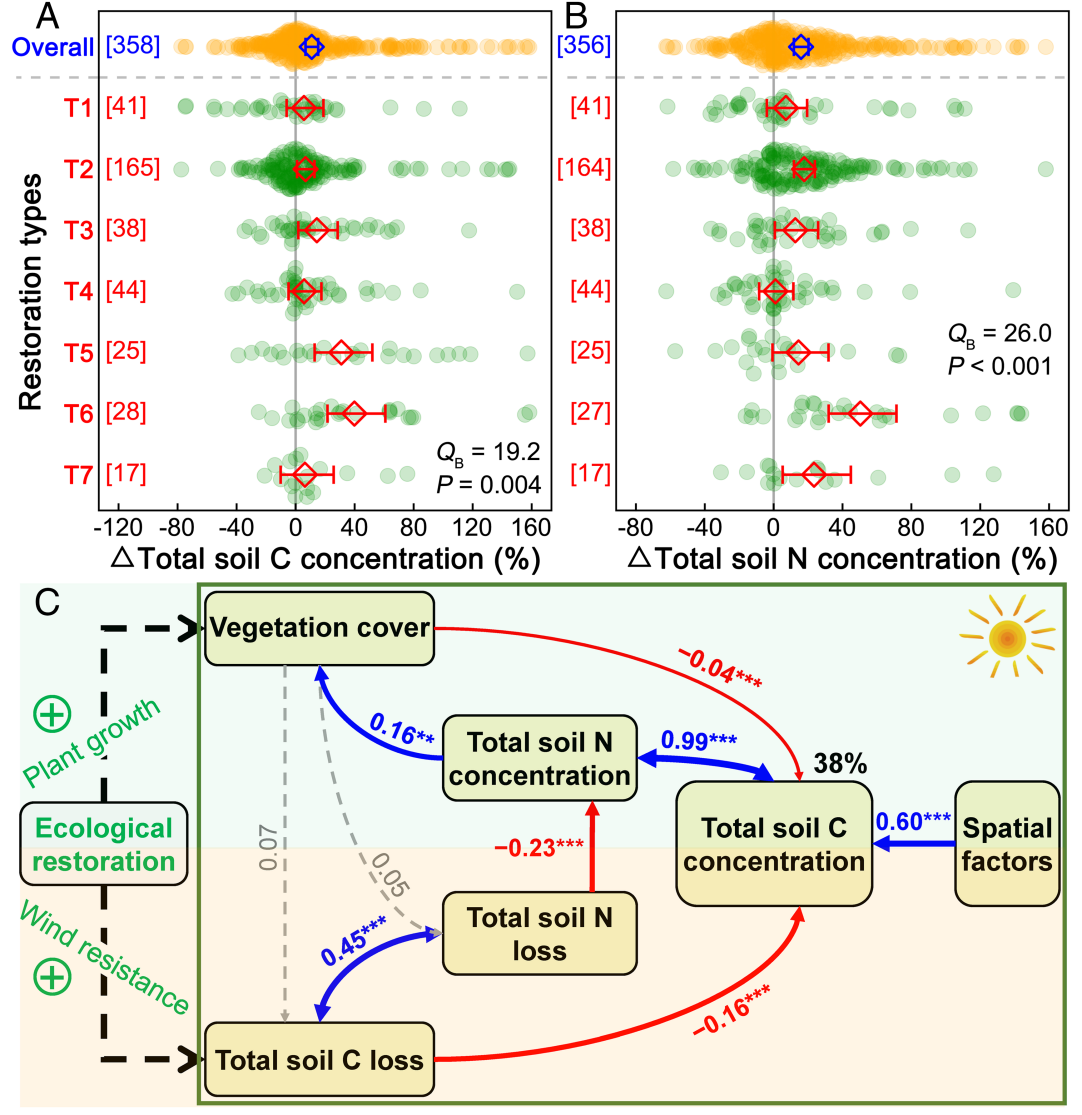
A regional survey demonstrates the impact pathways of ecological restoration that increase soil carbon (C) stocks. Percentage changes (mean ± 95% CI, diamond with error bar) in total soil C (*A*) and nitrogen (N; *B*) concentrations averaged across all (Overall) and in each of the seven ecological restoration types (T1−T7; Fig. 1). There is significant change if the 95% CI does not overlap zero. Colored scatter plots represent changes in these variables at each site. Significant between-group heterogeneity (*Q*_B_; *P* < 0.05) indicates that the percentage changes differ among the seven restoration types. The values in square brackets along the y-axis show sample size (number of sites). The optimal structural equation model (*χ*^2^ = 0.582, *df* = 2, *P* = 0.747, GFI = 0.999, RMSEA < 0.001) reveals all plausible pathways through which ecological restoration influences total soil C concentration (*C*). Red and blue solid arrows indicate significantly negative and positive pathways, respectively, and gray dashed arrows represent insignificant pathways. Numbers next to the arrows are standardized path coefficients with different levels of significance: **P < 0.01 and ***P < 0.001. The percentage value refers to the proportion of variance explained by the model.

Compared to the degraded lands, suppressions of wind erosion-induced losses can enhance soil C and N contents in the restored lands. Averaged across all the plot pairs, total soil C and N concentrations were 10.9% (95% CI: 6.7 to 15.3%) and 15.9% (11.8 to 20.2%) greater in restored than degraded lands, respectively (Fig. 2 *A* and *B*). This confirms the potential of ecological restoration in enhancing terrestrial C stocks and mitigating climate change (9, 34, 35, 47). Despite that all the seven restoration types exhibited positive effects on both total soil C and N concentrations, greater stimulations were observed under the conversion of croplands to natural grasslands or old-fields, as well as in the entire cropland category (*SI Appendix*, Fig. S9). This result is supported by findings from a 29-y field experiment conducted in the North Central United States (48). This phenomenon could be explained by the intensive disturbance caused by agriculture activities on top-soil profile, which leaves large area of bare soil with low cohesion and resistance, thus vulnerable to wind erosion and resulting in greater soil C loss during winter and spring compared to grasslands. In contrast, the slight increases in total soil C and N concentrations under desertification control measures may be attributed to the widespread presence of biological crusts in desert ecosystems, which could substantially enhance the cohesion of surface soils and their resistance to wind erosion and thus inhibit the losses of C and N in degraded areas (49). Given the widely documented N limitation in the terrestrial biosphere (50–52), greater soil N content and availability under ecological restoration can subsequently stimulate vegetation growth, cover, and photosynthetic C uptake, which accelerate the recovery of degraded ecosystems (53, 54) and finally enhance ecosystem C stocks and sequestration potential in drylands.

Based on the regional survey data, we first distinguished between the roles of ecological (increasing plant C input) and biophysical (reducing wind erosion C loss) pathways in regulating dryland soil C stocks during ecological restoration. In line with the well-documented positive relationships of plant growth and C uptake with soil N availability, our optimal SEM showed that increased soil N content due to reduced soil N loss under wind erosion in the restored ecosystems could in turn enhance vegetation cover (Fig. 2*C*). More surprisingly, however, suppressions of total soil C loss (the path coefficient: −0.16, *P* < 0.001, biophysical pathway), rather than the stimulated plant growth and vegetation cover (the path coefficient: −0.04, *P* < 0.001, ecological pathway), were predominately responsible for greater soil C contents under ecological restoration.

Soil C pools account for the majority of C stocks in the terrestrial ecosystems (55). Compared to the degraded lands, the biophysically conserved soil C content, resulting from the reduction of wind erosion-induced C loss, is much larger than the ecologically enhanced soil C content via stimulating plant growth and photosynthetic C uptake in the restored lands. This is supported by a previous work showing that changes in dryland plant growth dynamics have minimal impact on soil C stocks (35). These results align with a global observation of negative correlations between plant biomass and soil C responses to CO_2_ fertilization (56), underscoring that plant-soil C trade-offs may prevail in terrestrial ecosystems when exposed to natural and/or anthropogenic disturbances. Two mechanisms could help to explain this mismatch. On the one hand, ecological restoration may accelerate decomposition of soil organic matter to promote soil nutrient availability, because the sustained stimulations in plant growth under restoration require more nutrients (e.g., ref. 57). On the other hand, stimulated plant growth could increase new C inputs into soils, resulting in soil priming effects (58, 59). Both processes may enhance soil CO_2_ efflux, counterbalancing the positive effects of increased plant C inputs on soil C stock (60).

Our observations provide direct evidence that ecological restoration could enhance regional C stocks primarily by reducing wind erosion-induced soil C losses through dust emissions (see also refs. 42, 55). However, wind erosion and dust emissions have often been overlooked in most previous evaluations of ecosystem C budgets, despite their importance in wind erosion-prone drylands (28, 42). Our findings highlight the urgent need to include surface soil dynamics in land surface models to improve robustness of global C inventories (42). In addition to alterations in soil C loss and vegetation cover, random forest analyses demonstrated that local climate conditions, namely precipitation and temperature, could also play critical roles in shaping spatial variations of dryland soil C stocks under ecological restoration (*SI Appendix*, Fig. S10*A*). For instance, greater mean annual precipitation typically enhanced the positive effects of ecological restoration on soil C stocks at local scale, whereas increasing mean and maximum annual air temperature exhibited negative impacts (*SI Appendix*, Fig. S10 *B–D*). Both precipitation and temperature can influence soil moisture and vegetation cover, thereby affecting the resistance of surface soil to wind erosion (45). This result underscores the importance of taking local climate conditions into consideration in assessing spatial patterns in dryland C stocks under ecological restoration.

## A Decade-Long Manipulative Experiment

**Fig. 3.**
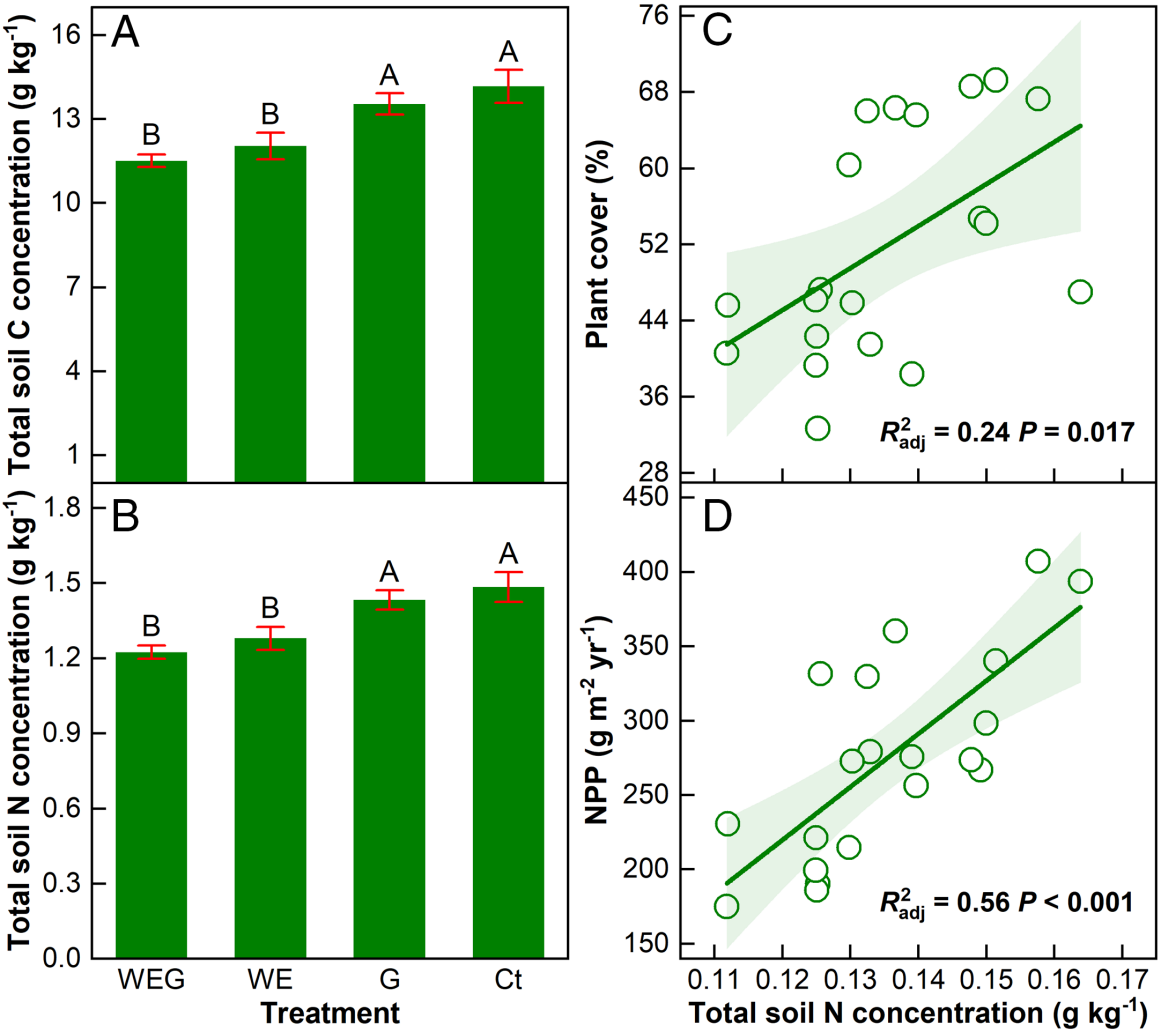
A 13-y experiment shows increased total soil carbon (C) and nitrogen (N) concentrations under ecological restoration. Mean (± 1 SE, *n* = 5) annual total soil C (*A*) and N (*B*) concentrations under the degraded (WEG, WE, and G) and restored (Ct) treatments. Different capital letters above the bars indicate significant differences among the four treatments. Spatial dependencies of plant cover (*C*) and net primary productivity (NPP; *D*) on total soil N concentration. Each data point represents 13-y mean value of each plot. WEG: wind erosion plus grazing, WE: wind erosion only, G: grazing only, and Ct: control.

We further used a dataset from a 13-y (2010–2022) manipulative experiment with grazing and/or simulated wind erosion (61) to validate and reveal explicitly the underlying mechanisms for the above findings of the comprehensive regional survey. In this experiment, grazing (G), wind erosion (WE), and wind erosion plus grazing (WEG) treatments were considered as slight (disturbing plants only), moderate (disturbing soil only), and severe degradations (disturbing both plants and soil), respectively, while the control treatment (Ct) served as a proxy for restored ecosystems (*Methods*). The annually mimicked wind erosion resulted in average losses of 12.2 ± 0.69 and 1.30 ± 0.07 g m^−2^ y^−1^ of total soil C and N, respectively, from the WE and WEG plots (*SI Appendix*, Fig. S11 *A* and *B*) whereas the same amounts of total soil C and N were preserved in the Ct and G plots. Averaged over the 13-y experimental period and the three degradation intensities, ecological restoration increased total soil C concentration by an average of 15.3 ± 3.3% (Fig. 3*A*), comparable to our observations in the regional survey (10.9%, Fig. 2*A*). In addition, the enhancements of total soil C concentration increased with the initial intensity of degradation, being 4.5 ± 2.4% from slight (Ct−G; *P* > 0.05) to 17.8 ± 3.4% from moderate (Ct−WE; *P* < 0.05) and 23.5 ± 6.9% from severe (Ct−WEG; *P* < 0.05) degradations. Moreover, the stimulation of total soil C concentration also intensified with the duration of ecological restoration (*SI Appendix*, Fig. S12).

**Fig. 4.**
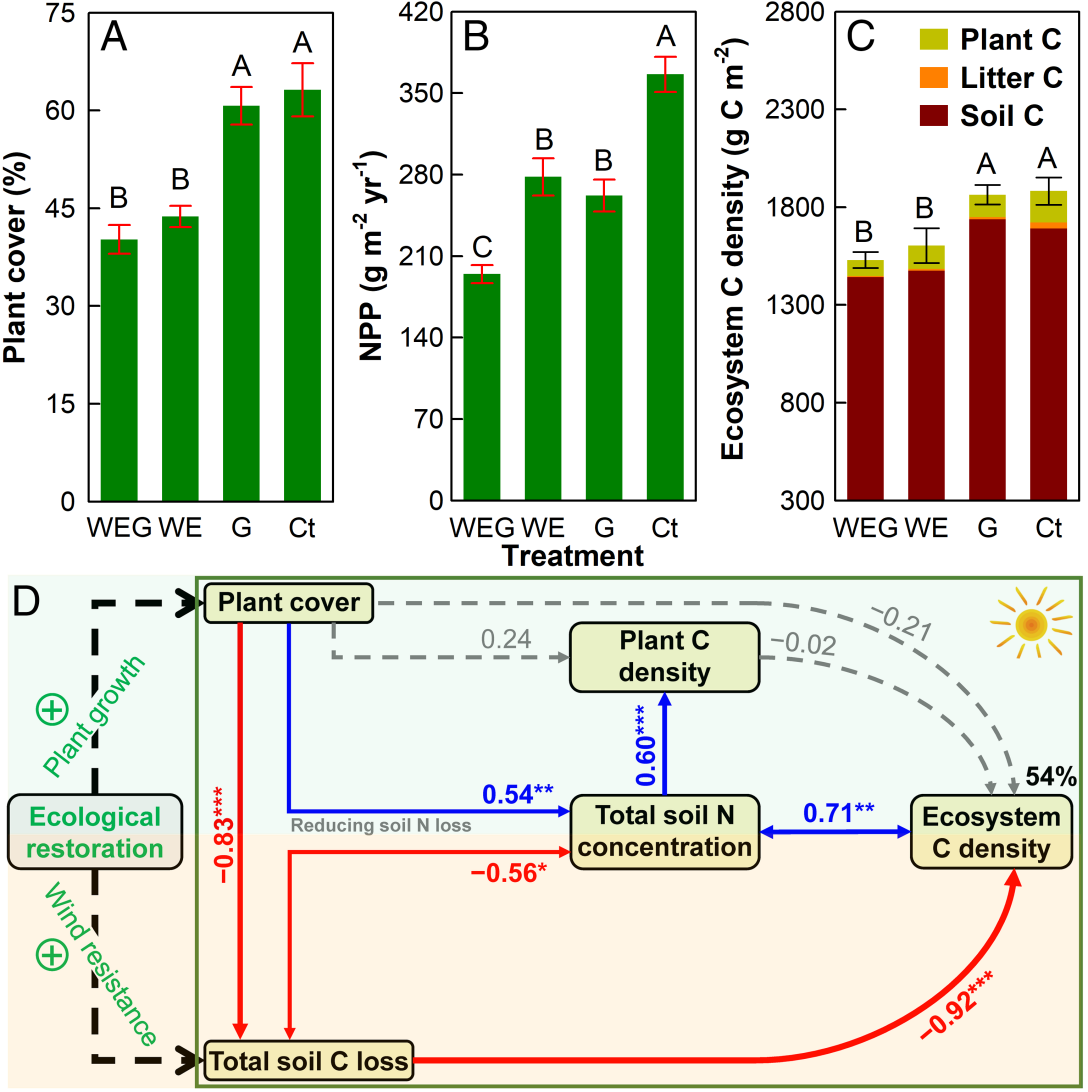
Pathways of ecological restoration enhance ecosystem carbon (C) stocks: Evidence from the decade-long experiment. Mean (± 1 SE, *n* = 5) annual plant cover (*A*), net primary productivity (NPP; *B*), and ecosystem C density (*C*) under the degraded (WEG, WE, and G) and restored (Ct) treatments. The optimal structural equation model (*χ*^2^ = 0.551, *df* = 1, *P* = 0.458, GFI = 0.989, RMSEA < 0.001) reveals all plausible pathways, including plant variables and total soil C loss, which affect ecosystem C density (*D*). Red and blue solid arrows indicate significantly negative and positive pathways, respectively, and gray dashed arrows represent insignificant pathways. Numbers next to the arrows are standardized path coefficients with different levels of significance: *P < 0.05, **P < 0.01, and ***P < 0.001. The percentage value refers to the proportion of variance explained by the model. Fig. 3 for abbreviations.

Due to the conservation role, total soil N concentration was promoted by 13.7 ± 3.1% under ecological restoration (Fig. 3*B*), and the positive effects increased with both degradation intensity (Ct−G, +3.5 ± 2.6%; Ct−WE, +16.0 ± 2.8%; Ct−WEG, +21.7 ± 6.6%) and restoration duration over the 13-y experimental period (*SI Appendix*, Fig. S13). Similarly, soil N availability was, on average, 21.2 ± 4.3% higher under restoration than degradation over the last five years from 2018 to 2022 (*SI Appendix*, Fig. S11*C*). Enhanced soil N contents under ecological restoration can stimulate plant cover (Fig. 3*C*; *R*^2^ = 0.24, *P* = 0.017) and net primary productivity (NPP; Fig. 3*D*; *R*^2^ = 0.56, *P* < 0.001). Random forest analyses further revealed that soil N availability and contents were the most important factors influencing plant cover and NPP, respectively, in this temperate steppe (*SI Appendix*, Fig. S14). Averaged across the 13-y experimental period, plant cover was 2.9% (absolute change, *P* > 0.05), 19.3% (*P* < 0.05), and 22.0% (*P* < 0.05) greater in the Ct than G, WE, and WEG plots, respectively (Fig. 4*A*), which is in agreement with the extensive observations in our regional survey (Fig. 1*C*) and previous studies (23, 39). In addition, NPP was 41.3 ± 9.2%, 33.8 ± 10.0%, and 89.4 ± 11.2% higher under the Ct than G, WE, and WEG treatments, respectively (Fig. 4*B*, all *P* < 0.05). Moreover, the increments of NPP also amplified with the restoration duration (*SI Appendix*, Fig. S15). As a consequence of the above-mentioned processes, it is reasonable to expect that enhanced plant growth will subsequently increase ecosystem C stocks.

We estimated ecosystem C density by summarizing the topsoil (0 to 10 cm), plant litter, and living biomass (*Methods*). The results showed that ecological restoration significantly enhanced ecosystem C density, on average, by 14.2 ± 3.6% (Fig. 4*C*) and the positive effects increased with the initial intensity of degradations (Ct−G: +0.88 ± 1.1%, *P* > 0.05; Ct−WE: +18.1 ± 4.4%, *P* < 0.05; and Ct−WEG: +23.6 ± 6.7%, *P* < 0.05). These experimental observations provide an additional line of evidence that ecological restoration promotes ecosystem C stocks in degraded lands, as observed in our regional transect survey in this work (Fig. 2*A*) and our previous regional survey of China’s grasslands conducted in 2009 (35, *SI Appendix*, Fig. S16), in North America (62), and around the world (47).

The optimal SEM based on these experimental data explained 54.0% of the variations in ecosystem C density under ecological restoration (Fig. 4*D*). Greater soil N concentration resulting from lowered N loss significantly stimulated plant C density in the restored plots (the path coefficient: 0.60, *P* < 0.001), in agreement with that enhanced soil N availability and contents under ecological restoration may promote plant growth and C uptake (Figs. 3 *C* and *D* and 4 *A* and *B*). Most importantly, the changes in total soil C loss were the dominant factor determining the variations in ecosystem C density (the path coefficient: −0.92, *P* < 0.001), whereas changes in either plant cover or C density had no effect on it (the path coefficient: −0.02, *P* > 0.05). These results provide site-scale support for the findings of our comprehensive regional survey and may be largely accounted for by that soil C density accounts for about 90% of ecosystem C density in this semiarid grassland (Fig. 4*C*).

Our decade-long manipulative experiment involving grazing and wind erosion treatments, alone or in combination, provides a unique opportunity to distinguish between the relative predictive power of ecological (increasing plant C input) and biophysical (reducing wind erosion C loss) pathways to variations in grassland C stocks. We found that the summed effects of increased plant C input (Ct−G: +0.62 g C kg^−1^ y^−1^) and reduced litter and soil losses (Ct−WE: +2.12 g C kg^−1^ y^−1^) on total soil C concentration were approximately equal to the observed influences of the two pathways in combination (Ct−WEG: +2.65 g C kg^−1^ y^−1^), suggesting that these analyses were acceptable and reliable. The reduced litter and soil losses accounted for 80.0% (estimated as $\frac{\mathrm{Ct}-\mathrm{WE}}{\mathrm{Ct}-\mathrm{WEG}}$) of the increases in total soil C concentration when both plants and soils were undisturbed, confirming our findings of the predominant role of the biophysical pathway in enhancing C stocks in the restored compared to degraded ecosystems. Similar to soil C content, decreased litter and soil losses accounted for 78.9% of the stimulated ecosystem C density under ecological restoration. Furthermore, the greater observed enhancements (Ct−WEG; +352 g C m^−2^) in ecosystem C density than the predicted increases ([Ct−G] + [Ct−WE]; +297 g C m^−2^) under the no-plant or no-soil disturbance scenarios can be explained by the positive feedback of soil nutrients to plant growth, supporting our SEM results based on the experimental data (Fig. 4*D*).

We further evaluated the significance of increased C sequestration through the biophysical pathway in China’s terrestrial C cycle. The incremental rates of soil C stocks due to reduced wind erosion C loss under ecological restoration in North and Northwest China were estimated to be 7.87 Tg C y^−1^ (*Methods*), contributing to 10.6% of enhanced land C sink (74.0 Tg C y^−1^) in China under national ecological restoration projects (34), to which afforestation contributed to 20.3 Tg C y^−1^ and forest protection (natural forest protection programs) contributed to 14.0 Tg C y^−1^. The increments (i.e., 7.87 Tg C y^−1^) could account for 0.64−0.68% of land net CO_2_ sink (−1,151 ~ −1,229 Tg CO_2_ y^−1^) and 10.5 to 27.1% of net greenhouse gas sink (−29.0 ~ −75.3 Tg CO_2_-eq y^−1^) and offset 1.81% of terrestrial CO_2_ emissions into the atmosphere (435 Tg CO_2_ y^−1^) in China during the 2000s and 2010s (63), representing a non-negligible component in national C sink inventories. These lines of evidence further underscore that neglecting the reduced C and nutrient losses under suppressed wind erosion in most previous studies may have significantly underestimated the C sink potential of drylands under ecological restoration.

## Implications for Conservations of Global Dryland Soil C by Ecological Restoration

In the context of C neutrality and climate change mitigation, it is of fundamental importance to accurately assess terrestrial C budgets and to generate robust global C inventories under various land use scenarios (1). Enhanced terrestrial C sink through ecological restoration is always intuitively and unanimously assumed to result from stimulated photosynthetic C fixation and plant growth (4–6). Surprisingly, our observations from both the comprehensive regional survey and the manipulative experiment clearly deny these conventional intuitions but demonstrate the dominant role of reduced surface soil C loss due to suppressed wind erosion in restoring dryland C stock. However, most land surface models used to evaluate land-based C mitigation strategies do not account for soil C loss caused by wind erosion in dryland ecosystems (42). Nevertheless, vegetation cover is relatively sparse due to low precipitation and water availability, C is mainly stored in the soil, and the surface soil is highly susceptible to wind erosion in drylands (13, 19, 45). Therefore, dryland surface soils represent a significant fraction of labile C that is vulnerable to decomposition in the terrestrial C cycle and a global source of atmospheric CO_2_ (64).

The lateral transfer, redeposition, and fixation of wind-blown dust, which are not considered in this study, may serve as important uncertainties in accurately estimating regional C stocks. In contrast to the hypothesis that a significant portion of C released during wind erosion is lost due to oxidation, findings from a pioneering study conducted across the United States have revealed that approximately 33% of wind-eroded soil organic C redeposits locally (65). However, consistent with the wind-eroded C lost hypothesis, data from our 13-y manipulative experiment (61) provide evidence that dust deposition does not significantly affect total soil C concentration in the temperate steppe (*SI Appendix*, Fig. S17*A*). This lack of positive impacts of dust deposition may be attributed to soil priming effects induced by new C inputs and accelerated decomposition of old C (58), as evidenced by the increased soil CO_2_ emissions in the dust-deposited plots (*SI Appendix*, Fig. S17*B*). Thus, it could help to address potential concerns about the uncertain impacts of redeposited soil on local soil C content. We have clearly quantified the relative contributions of ecological and biophysical pathways to enhanced ecosystem C stocks in the source region of wind erosion under land restoration. Our findings explicitly emphasize the importance of the largely ignored benefits of ecological restoration in most of previous studies, i.e., reducing wind erosion and C and nutrient losses. This work provides a particularly strong case from China and reveals a unique but always-overlooked mechanism for restoring vast drylands to enhance terrestrial C stock. Global assessments of the significant importance of conserving and increasing the stability and C storage of surface soils in the wind erosion-prone regions may be as important in mitigating rising atmospheric CO_2_ concentrations and climate change as enhancing forest C sinks (e.g., ref. 66).

## Methods

### Large-Scale Regional Surveys.

Our in situ regional surveys were conducted in North and Northwest China covering five provinces including Inner Mongolia, Ningxia, Gansu, Qinghai, and Xinjiang and ranging from 36.05°N to 48.20°N and 76.30°E to 117.52°E (Fig. 1*A*). Semiarid, arid, and hyperarid ecosystems are prevailing in these regions and represent one of the typical landscapes in Eurasia and across other broad geographical ranges. High wind speeds and low precipitation lead to North and Northwest China becoming important sources of dust emissions globally (27, 43), with few instances of surface runoff and water erosion (*SI Appendix*, Fig. S1 and Table S2). Over the second half of the 20th century, these regions have experienced extensive land degradation and wind erosion mainly due to overgrazing and agricultural reclamation (23, 67) and represented one of the global priority regions for ecological restoration (68). The Central Government of China has implemented 14 ambitious state-sponsored ecological projects since the 1970s to combat land degradation and wind erosion and 8 out of the 14 programs have covered these regions (23). Specifically, the grassland conservation and restoration programs have been implemented across over a million square kilometers during the past two decades (*SI Appendix*, Fig. S18). These projects provide a unique opportunity to comprehensively investigate the possible benefits of ecological restoration in enhancing regional carbon (C) sink.

In the spring and autumn of 2014–2016, when most wind erosion and dust storm events occur (46), we conducted four field transect surveys simulating wind erosion in situ across the five provinces (Fig. 1*A* and *SI Appendix*, Fig. S2). In Apr.−May 2014, 1902 plots at 101 sites in Inner Mongolia were surveyed, and pairwise sampling was conducted at 44 sites with 558 plot pairs (degraded vs restored; *SI Appendix*, Table S1). In the second transect survey in Sep.−Oct. 2014, 246 pairs of plots at 82 sites in Inner Mongolia were sampled. In the third transect survey in Apr.−Jun. 2015, 12 plots at 2 sites in Gansu, 670 plots at 126 sites in Xinjiang, and 57 plots at 13 sites in Qinghai were sampled, with 2 (6 plot pairs), 88 (264 plot pairs), and 1 (3 plot pairs) sites using pairwise sampling in Gansu, Xinjiang, and Qinghai, respectively. In Apr.−Jun. 2016, we sampled 801 plots at 135 sites in Inner Mongolia, 30 plots at 5 sites in Ningxia, and 315 plots at 53 sites in Gansu in the last survey, having 117 (351 plot pairs), 5 (15 plot pairs), and 52 (156 plot pairs) sites using pairwise sampling in Inner Mongolia, Ningxia, and Gansu, respectively. In total, there were 4279 1 × 1 m^2^ plots across 517 sites, including 1599 plot pairs (3198 plots) at 391 of these sites. Each site had at least 3 replicates, i.e., plots at nonpair sampling sites and plot pairs at pair sampling sites.

First, the two plots in each randomly located pair were carefully selected to have the same slope and elevation and the distance between the two plots was approximately 10 m to minimize the confounding impacts of spatial heterogeneity on the evaluation of the restoration effects (Restored minus Degraded; *SI Appendix*, Fig. S2). Second, in order to cover the area and represent the general conditions at each sampling site, the distance between any two adjacent plot pairs (i.e., replicates) ranged from over 50 m to less than 1 kilometer. Third, given the vast dryland regions in North and Northwest China, each sampling site was randomly selected along the roads in the survey region with the distance between any two adjacent sites larger than 50 km with an attempt to capture the spatial heterogeneity across various landscapes or ecosystem types (grasslands, Gobi, deserts, and farmlands, etc.) in the more than three million km^2^ of dryland area.

Based on the strategies of China’s ecological engineering such as the conversion of croplands to forests or grasslands, grazing prohibition and rodent pest control, and setting straw checkerboards in deserts (23, 69), the 1599 plot pairs were classified into seven types (T) of ecological restoration (i.e., the adjacent degraded vs. restored vegetation pairs), including T1: grazed/degraded vs. ungrazed/undisturbed grasslands, T2: bare lands vs. disturbed/undisturbed grasslands/shrublands, T3: poorly developed vs. well-developed grasslands/shrublands, T4: deserts without vs. with desertification control, T5: croplands vs. natural grasslands, T6: croplands vs. old-field grasslands, and T7: bare soil vs. crops/no tillage with residue retention. The T1−T4 refer to grassland conservation and restoration as well as desertification control, and the T5−T7 represent the conversion of traditional agriculture to old-field/natural grasslands or conservation agriculture (*SI Appendix*, Table S3).

The choice of the ecological restoration types at each site depended on the typical ecological restoration practices at the local area. The proportion of each type in the four transect surveys is shown in *SI Appendix*, Fig. S19. Except for the single crop field (monoculture of wheat, corn, or cotton in T5−T7), the vegetation in other samples was native grasslands or secondary grasslands (i.e., old-fields) or shrublands (*SI Appendix*, Table S3). In homogeneous landscapes such as grasslands, deserts, and croplands, 1 × 1 m^2^ plots provided a representative sampling method for the surrounding distribution of vegetation. However, in sparse shrublands, we selected areas where the proportion of individual cluster of shrubs and bare land closely matched the surrounding landscape to accurately assess the C sequestration benefits of ecological restoration. Furthermore, the Great Green Wall project has been documented to have side effects, such as the overconsumption of groundwater and unsustainable soil water supply due to large-scale tree planting, resulting in low survival rates of the planted trees in return (70). However, tree planting could lead to lower wind speeds and enhance surface roughness, both of which suppress wind erosion, dust emission, and soil loss. Therefore, including plot pairs from the Great Green Wall project in our broader-scale regional survey might not have biased our main conclusions and findings of restoration effects on dryland C stocks.

During the wind erosion simulations, a portable pneumatic extinguisher (Taining Machinery Ltd. Co., Taizhou, Jiangsu, China) with adjustable wind speeds was used to simulate wind erosion in situ by blowing the soil surface in each plot (*SI Appendix*, Fig. S2). The extinguisher was modified by field experimental tests prior to the large-scale field survey to precisely control the wind speed at the air outlet (*SI Appendix*, Fig. S20). During the processes of simulated wind erosion, one operator held the portable pneumatic extinguisher to blow the 1 × 1 m^2^ plot from one side for thirty seconds with the outlet less than 20 cm above the ground. One or two other team members put a wind-proof cloth bag at the other side of the plots and to collect plant litter and soil lost from the plot (*SI Appendix*, Figs. S2 and S20). Plant litter and soil collected were used to determine C and nutrient losses.

In the first transect survey, wind speeds of 12, 16, 21, 25, and 30 m s^−1^ were tested to determine the threshold for severe wind erosion on the soil surface. The results showed that a wind speed of 25 m s^−1^ was sufficient for the experimental design (*SI Appendix*, Fig. S3). Therefore, a wind speed of 25 m s^−1^ was employed in the subsequent three transect surveys. In addition, we measured ambient wind speeds during the field manipulation of wind erosion using an anemograph, with median wind speeds recorded at 5.5 m s^−1^ (*SI Appendix*, Fig. S5). Importantly, variations in ambient wind speed did not impact our estimates of plant litter and soil dry mass losses during simulated wind erosion. During the four transect surveys, there were at least three replicates (i.e., plots) for wind erosion simulations under each wind speed at each site. Plant litter and soil lost induced by the simulated wind erosion were collected using a wind-proof cloth bag attached to the pneumatic extinguisher to determine the extent of C and nutrient losses.

### The Decade-Long Manipulative Experiment.

A 13-y two-factor (grazing and aeolian processes) experiment has been conducted since April 2010 in a semiarid temperate steppe in Duolun County (42°02´N, 116°17´E, 1324 m a.s.l.), Inner Mongolia, China (Fig. 1*A* and *SI Appendix*, Fig. S2; ref. 61). A randomized complete block design was used with six treatments including control (Ct), wind erosion (WE), dust deposition (DD), grazing (G), WEG, and dust deposition plus grazing (DDG). There were five replicates for each treatment and thus thirty 4 × 4 m^2^ plots, with a 1.5-m buffer zone between any two adjacent plots. The effects of dust deposition were not included in this study. A 1-y-old sheep was used to forage for 4 h in each grazed plot per month from June to September each year. Similar to the methods in the regional survey, a portable pneumatic extinguisher was used to blow off a 1 cm layer of surface soil to mimic wind erosion in early May each year. Soil and litter lost were collected using a cloth bag during the wind erosion simulations to record the amount of soil loss from the wind-eroded plots.

### Measurements of Total Soil C and N Concentrations and Soil C Pool Estimates.

In the regional survey, two soil cores at a depth of 0 to 60 cm (5 cm in diameter; four layers: 0 to 10 cm, 10 to 20 cm, 20 to 40 cm, and 40 to 60 cm) were collected for part of the sites due to the difficulty (constrains of time and soil texture) in taking samples at all the above layers in all the plot pairs. To account for the potential “fertility islands” effects often observed in drylands (37), one core was taken between plant individuals and another near plant roots. There were 358, 122, 107, and 103 sites with soil samples from these four layers, respectively. Soil samples at each depth from these two cores were sieved using a 2-mm mesh to separate plant litter, and then mixed together to create a composite sample representing the plot. In the manipulative experiment, three soil cores (5 cm in diameter and 10 cm in depth) were collected from two opposite corners and the center of each plot in early August each year. The three soil samples were mixed to form a composite sample of the plot. Roots and organic debris were removed from the mixed samples with a 2-mm mesh. These soil samples were air-dried and ground. An element analyzer (Vario MACRO CUBE, Elementar Inc., Germany) was used to measure total C and N concentrations of the soil samples collected from both the regional survey and the manipulative experiment.

This study focused exclusively on surface soil carbon (0 to 10 cm) for two reasons. First, our analyses demonstrated neutral responses of subsoil (10 to 60 cm) C concentration to ecological restoration in the regional survey (*SI Appendix*, Fig. S21). Second, our previous study on the impacts of ecological restoration on grassland C sink potential revealed that the increase of soil C density is mainly observed in the upper 1 m of alpine meadows, where water availability is not a limiting factor (*SI Appendix*, Fig. S16). In the manipulative experiment, to estimate the size of soil C pool at a depth of 10 cm, the bulk density of the top 10 cm soil was determined using a standard container with a volume of 100 cm^3^ (5.5 cm in diameter and 5 cm in height). Rock fragments were removed from the bulk density samples using a 2-mm mesh. The gravimetrical water content of the bulk density samples was measured after desiccating for 48 h at 105°C. Soil bulk density was calculated as the ratio of the soil dry mass to the container volume. This measurement was used to convert total soil C concentration in g C kg^−1^ soil to soil C content in g C m^−2^.

### Measurements of Plant Growth and Productivity and Plant C Pool Estimates.

In both the regional survey and the manipulative experiment, the percent plant cover of each plot was visually estimated. In the manipulative experiment, plant aboveground parts and litter of two 1 × 0.15 m^2^ quadrats at two opposite corners of each plot were harvested at peak biomass in early August each year. The plant and litter samples were oven-dried at 65 °C to constant weight and weighed as aboveground net primary productivity (ANPP) and litter mass, respectively. The root ingrowth method was used to measure belowground net primary productivity (BNPP). In early May each year, two 50-cm deep cylindrical holes were excavated in each plot with a 7-cm-diameter soil auger. The soil was refilled to the same hole after removing roots and rocks via a 2 mm sieve. A 5-cm-diameter soil auger was used to collect root ingrowth samples at the center of the original root ingrowth holes in early October each year. The dry mass of roots was oven-dried at 65 °C to constant weight and weighed as BNPP. Net primary productivity (NPP) was calculated as ANPP plus BNPP.

We measured plant and litter C contents using an element analyzer (Vario MACRO CUBE, Elementar Inc., Germany) to estimate plant and litter C pools. In addition, ecosystem C pool was calculated by summing soil, plant, and litter C pools.

### Data Analyses.

Losses of total soil C and N induced by the simulated wind erosion in the regional survey were calculated using data of the amounts of soil dry mass loss and C and N concentrations of the collected surface soil. The effect sizes of ecological restoration were calculated to examine responses of the losses of plant litter, soil dry mass, total soil C, and total soil N as well as total soil C and N concentrations to ecological restoration averaged across all 1,599 degraded-and-restored plot pairs, under each of the five wind speeds in the first transect survey, as well as in different ecosystems (prior to ecological restoration) and under each of the seven types of ecological restoration exposed to 25 m s^−1^ wind erosion in all four transect surveys. The natural log-transformed response ratios (RRs) were used to estimate the effect size of ecological restoration:

[1] $$\ln RR=ln\left(\frac{\bar{X_{R}}}{\bar{X_{D}}}\right),$$

with a variance of:

[2] $$\mathrm{var}(\mathrm{RR})=\frac{{\mathrm{SD}}_{R}^{2}}{n_{R}{\bar{X}}_{R}^{2}}+\frac{{\mathrm{SD}}_{D}^{2}}{n_{D}{\bar{X}}_{D}^{2}},$$

where $\bar{X}$, SD, and *n* represent mean value, SD, and sample size of these variables in the three restored (R) and three degraded (D) plots under each wind speed at each site, respectively. We calculated the weighted response ratio (ln RR_++_) and bias-corrected 95% bootstrap-CI using the “metafor” package in R (51). In these analyses, we incorporated “sites” as random factors to meet the independence criteria for data use in meta-analysis. Percentage changes (%) in these variables induced by ecological restoration were evaluated as (*e*^lnRR++^ − 1) × 100. The responses of these variables to ecological restoration were considered to be statistically significant if the 95% CIs of the restoration impacts did not overlap with zero. In addition, paired *t* tests were used to examine the effects of ecological restoration on vegetation cover by comparing differences in these variables between plots without and with ecological restoration. The spatial dependences of the summed losses of plant litter and soil dry mass on vegetation cover were examined with exponential regression models using all data collected from the 517 sites. Simple linear regressions were used to explore the dependences of wind erosion-induced losses (represented by values of RRs) of plant litter, soil dry mass, total soil C, and total soil N on the changes in vegetation cover under ecological restoration using the data collected from the 391 sites with pairwise sampling. Multiple comparisons with LSD tests were performed to compare the differences in total soil C and N concentrations, plant cover, and soil and ecosystem C pools among the four treatments (i.e., Ct, WE, G, and WEG) in the manipulative experiment. All the data used in analyses of multiple comparisons were tested and met the criteria for normality. All the aforementioned statistical analyses were performed using R version 4.4.0.

We further used random forest algorithms to identify the primary predictors for the spatial patterns of the changes in wind erosion-induced plant litter, soil dry mass, total soil C, and total soil N losses, as well as the changes in total soil C concentration under ecological restoration. These predictors encompassed factors related to vegetation (cover and height), soil properties (soil moisture and bulk density), local climate conditions (mean annual precipitation and temperature), and geographical positions (latitude, longitude, and elevation). In random forests, classification trees with binary divisions were collected and the fit of each tree was evaluated using 1/3 of the randomly selected out-of-bag data. The importance of each variable was estimated by running the random forest 100 times and then was used to evaluate the increase in the mean square error. The most fitting values of “ntree,” “mtry,” and “nodesize” were set as 5,000, 3, and 5, respectively, based on the results of multiple computations. R package “randomforest” was performed to conduct these analyses. The *P* value of the importance of each variable was assessed using the R package “rfPermute.”

Based on the results of random forests, we examined the relative predictive power between the ecological (increasing plant C input) and biophysical (reducing wind erosion C loss) pathways to increased soil and ecosystem C stocks in both the regional survey and the decade-long manipulative experiment. Structural equation modeling (SEM) linking all plausible pathways through which ecological restoration affects ecosystem C dynamics was constructed using AMOS 17.0.2 (Amos Development Corporation). The chi-square (*χ*^2^; with *P* value greater than 0.05 indicating a good fit), GFI (goodness-of-fit index), and RMSEA (Rms error of approximation) tests were performed to evaluate the goodness of the model fits. The closer the GFI value is to 1 and the RMSEA value is to 0, the better the model fit. Before conducting the SEM, we used the “spdep” package in R to examine whether the data from the regional survey exhibited autocorrelation. The analyses revealed that, with the exception of total soil N concentration, the remaining four variables (vegetation cover, total soil C loss, total soil N loss, and total soil C concentration) demonstrated significant spatial autocorrelation. Therefore, we performed the “spdep,” “nlme,” and “lavaan” packages in R to construct spatial residuals. These residuals were incorporated into the SEM constructed using the regional survey data to address spatial factors and mitigate the impact of spatial autocorrelation on the evaluation of the SEM.

To evaluate the significance of the conserved C by the biophysical pathway in China’s terrestrial C cycling, we calculated the increments of soil C stocks induced by reducing wind erosion C loss under ecological restoration in North and Northwest China. First, we collected the peer-reviewed data from the ref. 34 on the increased C sink induced by ecological restoration projects—specifically those focusing on grassland conservation and sand control—implemented in North and Northwest China. The two restoration projects increased ecosystem C stocks by 80.9 (time period: 2003−2010, area: 60 × 10^6^ ha) and 69.7 Tg C (2001−2010, 3.3 × 10^6^ ha), respectively, in North and Northwest China, with enhancements of soil C sink by 39.2 and 12.3 Tg C, respectively. As a result of continuous efforts by the Chinese government, the protected area of grasslands reached 105 × 10^6^ ha in 2022 (national grassland monitoring report; https://www.forestry.gov.cn/). Therefore, we calculated the increasing rates of soil C sink under grassland conservation based on the data collected from ref. 34 as 0.082 Tg C y^−1^ 10^6^ ha^−1^. Based on our findings of approximately 80% of increased soil C concentrations resulting from reduced surface soil C loss due to wind erosion, we then estimated the increments of soil C stocks by the biophysical pathway under grassland conservation and sand control as (0.082 Tg C y^−1^ 10^6^ ha^−1^)× (105 × 10^6^ ha)×80% + 12.3 Tg C/10(year) × 80% = 7.87 Tg C y^−1^ in North and Northwest China.

## Supplementary Material

Appendix 01 (PDF)

Movie S1. Video on a sandy storm met by the team at 15:56 pm (Beijing Time) May 20, 2016 during the fourth transect survey.

## Data Availability

The data supporting the findings of this study as well as the R code used can be accessed by visiting the figshare link: https://doi.org/10.6084/m9.figshare.24902922 (71). The R code we used does not include any functions or packages that we created or modified. All study data are included in the article and/or *SI Appendix*.
