# Supplementary material for: Ecological restoration enhances dryland carbon stock by reducing surface soil carbon loss due to wind erosion
Source: Proc Natl Acad Sci U S A. 2024 Nov 8;121(46):e2416281121. doi: 10.1073/pnas.2416281121 (PMC11573679; doi:10.1073/pnas.2416281121)
Supplement: Supplementary file 1 — Appendix 01 (PDF) [file pnas.2416281121.sapp.pdf]

Supplementary Information for

Ecological restoration enhances dryland carbon stock by reducing surface soil carbon loss due to wind erosion

Jian Song[1], Shiqiang Wan[1,*], Kesheng Zhang[2], Songbai Hong[3], Jianyang Xia[4,5], Shilong Piao[6,7], Ying-Ping Wang[8], Jiquan Chen[9], Dafeng Hui[10], Yiqi Luo[11], Shuli Niu[12], Jingyi Ru[1], Hao Xu[6], Mengmei Zheng[13], Weixing Liu[14], Haidao Wang[1], Menghao Tan[1], Zhenxing Zhou[1], Jiayin Feng[1], and Xueli Qiu[1]

*To whom correspondence may be addressed. Email: swan@hbu.edu.cn.

This file includes:

- Supplementary Figures 1−21 (pp. 2-22)
- Supplementary Tables 1−3 (pp. 23-25)
- Legend for supplementary vedio (pp. 26)

Figure S1. Spatial patterns of wind speeds, total surface runoff, and soil erosion. Global patterns of wind speeds (*A*) and total surface runoff (*B*) over 2010−2014 simulated by Beijing Climate Center's Earth System Model (BCC-CSM2-MR). Soil erosion map across China's drylands (*C*), constructed based on "Spatial Distribution of Soil Erosion in China" from the Chinese Academy of Sciences Resource and Environment Science Data Center, with the locations of sampling sites of regional survey and the field experiment site in Duolun County.

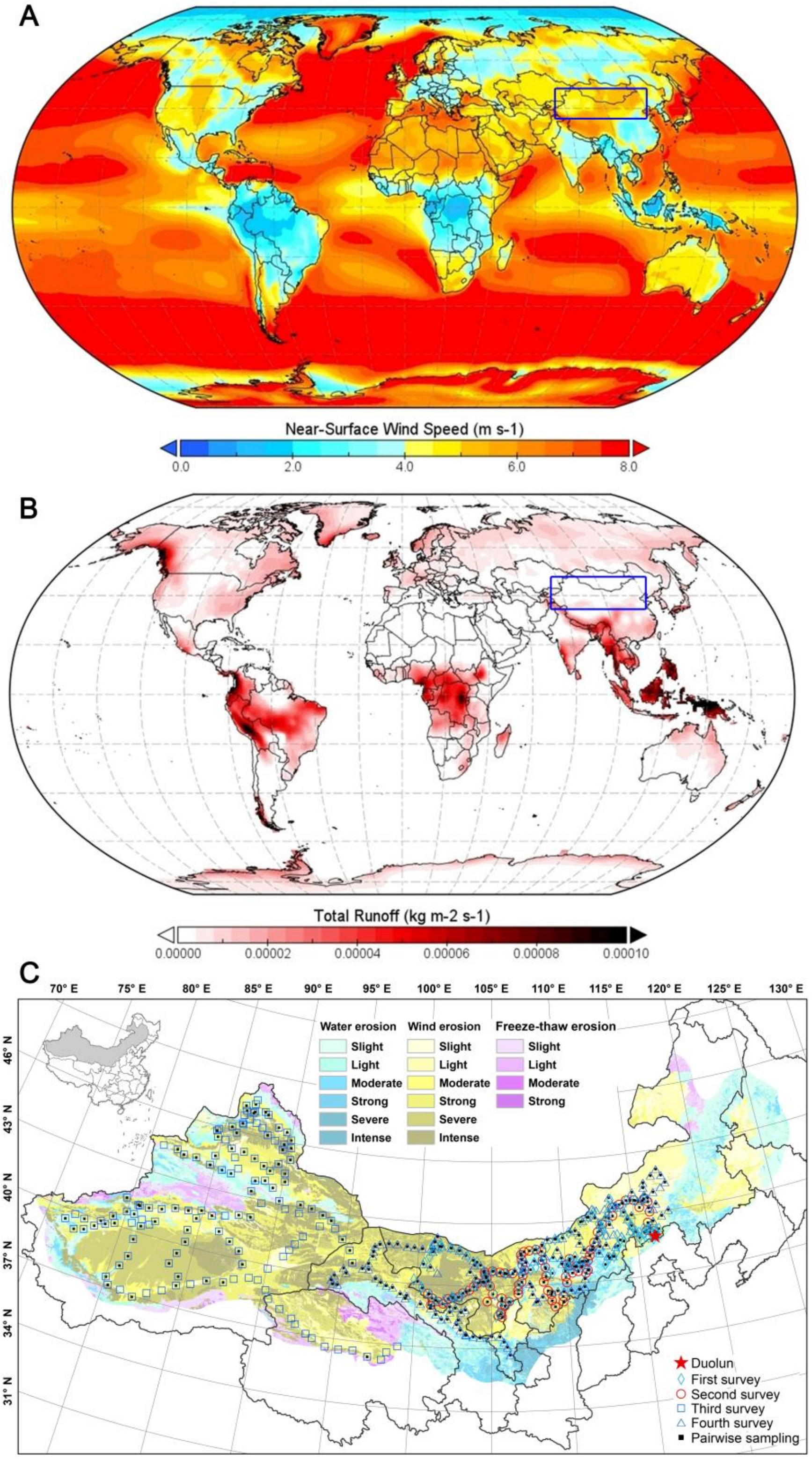

Figure S2. The revised pneumatic extinguisher with adjustable wind speeds to mimic wind erosion *in situ*. The vertical section viewport (*A*) and horizontal section viewport (*B*) of *in situ* wind erosion simulator used in this study. Note: 1: soil, 2: a wind-proof cloth bag in front of the simulator to collect plant litter and soil, 3: polyvinyl chloride boards around the sampled plot, 4: cylindrical ruler, 5: positioning telescopic component, 6: a modified portable pneumatic extinguisher, 7: baffle plate, 8: the sampled plot, and 9: polyvinyl chloride boards around the sampled plot. Patent number: ZL 2017 2 0218674.8 (China National Intellectual Property Administration; see also Figure S19*F*). *In situ* simulation of wind erosion in the experimental plots (*C*), a desert (*D*), and a Gobi (*E*). *In situ* dust storm event met by the team on the road of Taklamakan Desert (*F*). The schematic diagram of pairwise sampling design (*G*); First, the two plots within each randomly-located pair were carefully selected to have the same slope and elevation and the distance between the two plots was approximately 10 m to minimize the confounding impacts of spatial heterogeneity on the evaluation of the restoration effects (Restored minus Degraded). Second, in order to cover the area and represent the general conditions at each sampling site, the distance between any two adjacent plot pairs (i.e., replicates) ranged from over 50 meters to less than 1 kilometer. Third, given the vast area of the drylands in North and Northwest China, each sampling site was randomly selected along the roads in the survey region with the distance between any two adjacent sites larger than 50 km with an attempt to capture the spatial heterogeneity across various landscapes or ecosystem types (grasslands, Gobi, deserts, and farmlands etc.) in the more than three million km^2 of sampling area.

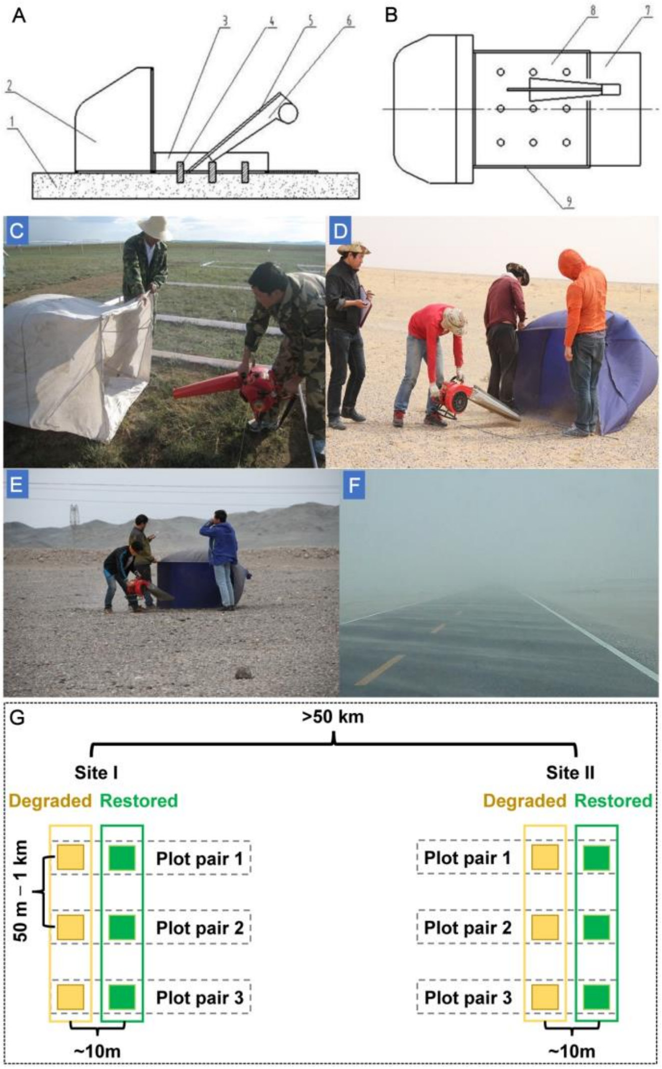

Figure S3. Plant litter and soil dry mass losses along a wind speed gradient. Losses of plant litter plus soil dry mass (*A*) and soil dry mass alone (*B*) under simulated wind erosion with five wind speeds of 12, 16, 21, 25, and 30 m s^{-1}. The red solid lines in the boxes and the "+" above the boxes are the means and outliers, respectively.

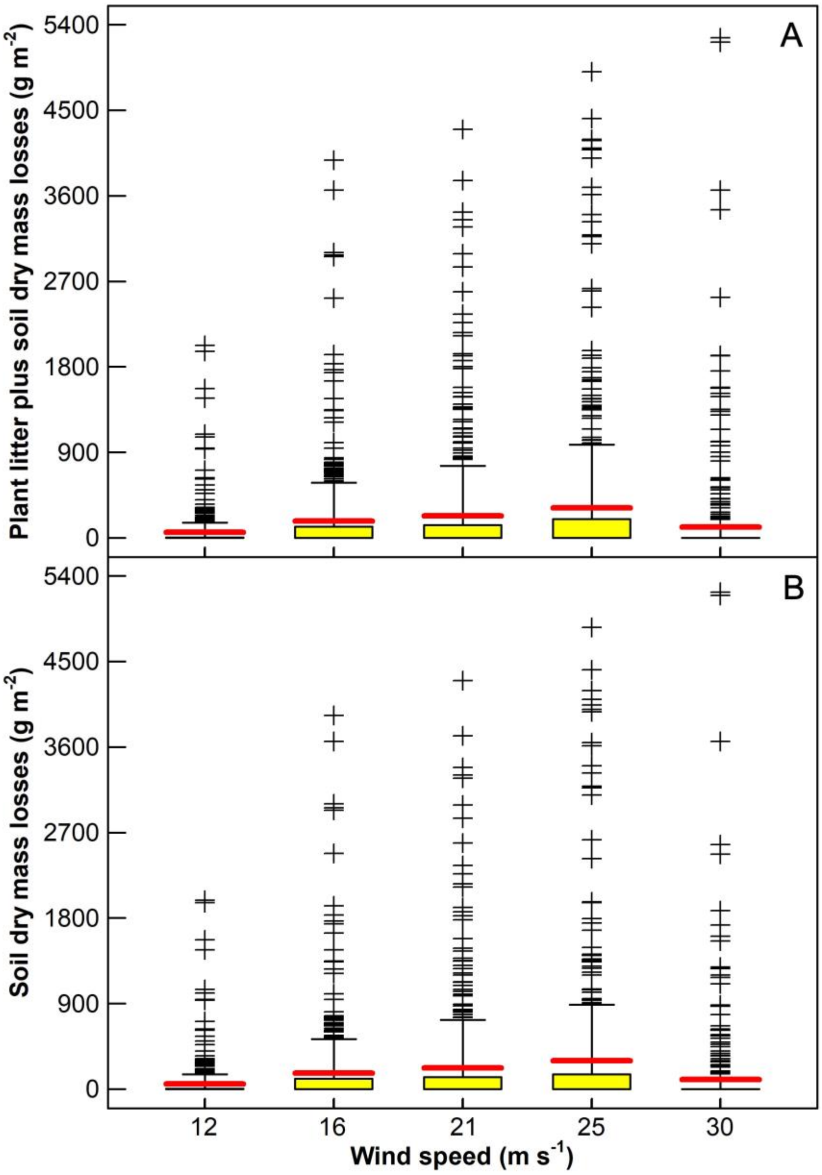

Figure S4. Losses of plant litter and soil dry mass under simulated wind erosion with five wind speeds. Ecological restoration-induced percentage changes (mean ± 95% CI, diamond with error bar) in the losses of plant litter (*A*), soil dry mass (*B*), total soil carbon (C; *C*), and total soil nitrogen (N; *D*) under wind erosion with wind speeds of 12, 16, 21, 25, and 30 m s^{-1} during the first field survey in spring 2014. Colored scatter plots represent percentage changes at each site. There is significant change if the 95% CI does not overlap zero. Significant between-group heterogeneity (Q_B; $P < 0.05$) indicates that the percentage changes differ among the five wind speeds. The blue values in square brackets show sample size (number of sites).

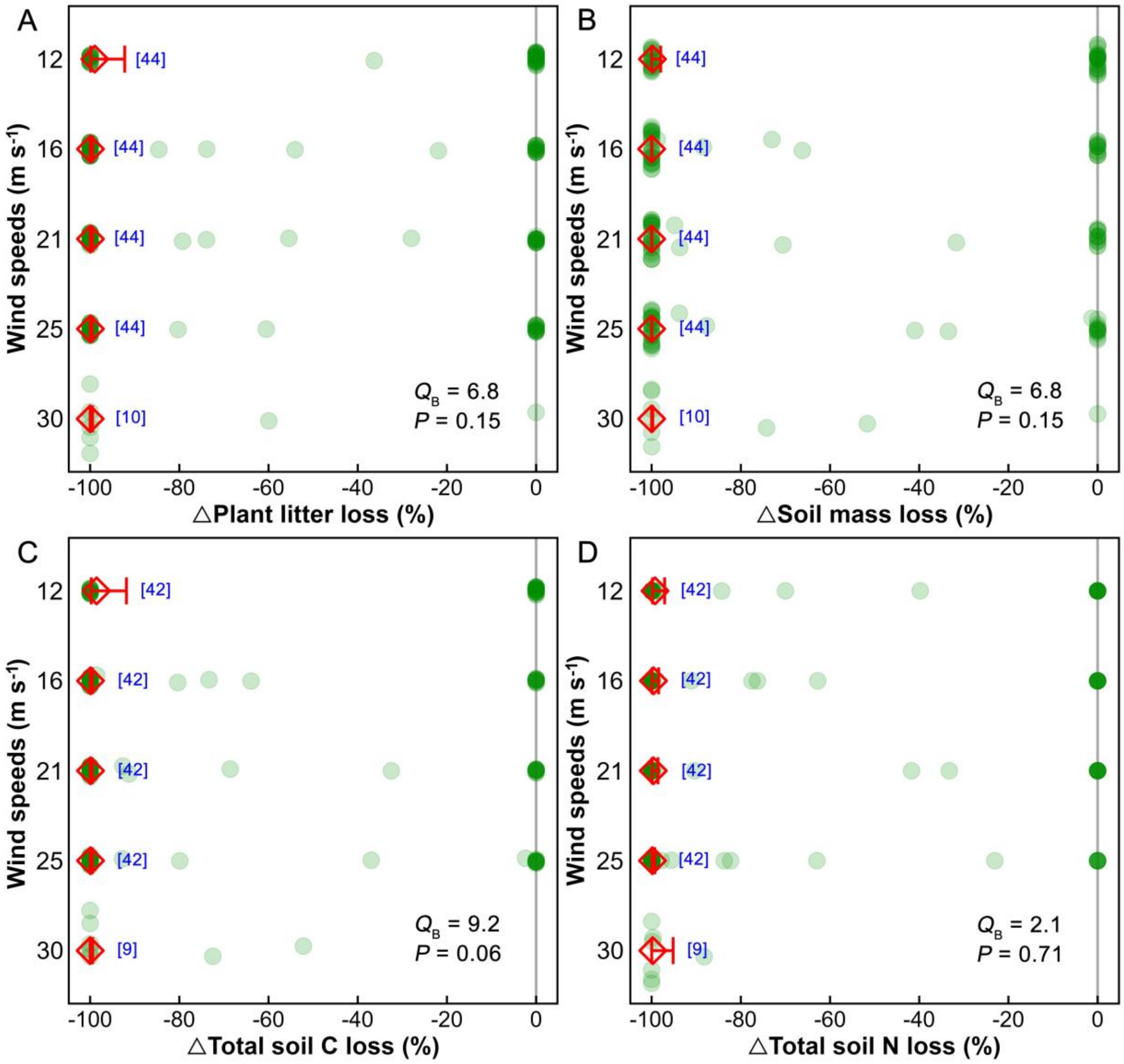

Figure S5. Effects of ambient wind speeds. Ambient wind speeds measured at 255 of the 391 paired sampling sites (*A*). Relationships of ecological restoration-induced changes in plant litter plus soil dry mass losses (*B*) and soil dry mass losses (*C*) with ambient wind speeds. Each data point represents mean value (± 1 SE) at each site. Red dashed lines represent insignificant linear regressions.

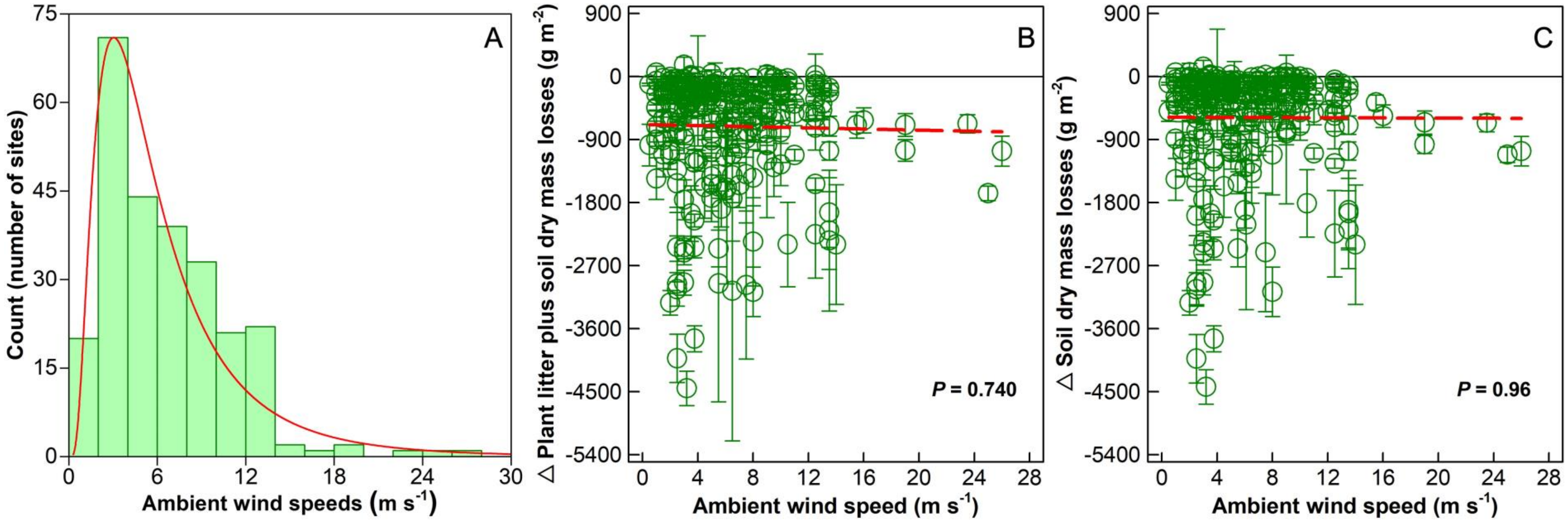

Figure S6. The attenuation of the manipulated wind speeds. Declining wind speed with increasing distance from the pneumatic extinguisher outlet at wind speeds of 16.5−16.7 m s^{-1} (*A*) and 20−22 m s^{-1} (*B*) in the test plots without (green dots) and with shrubs (red dots) in the first transect survey.

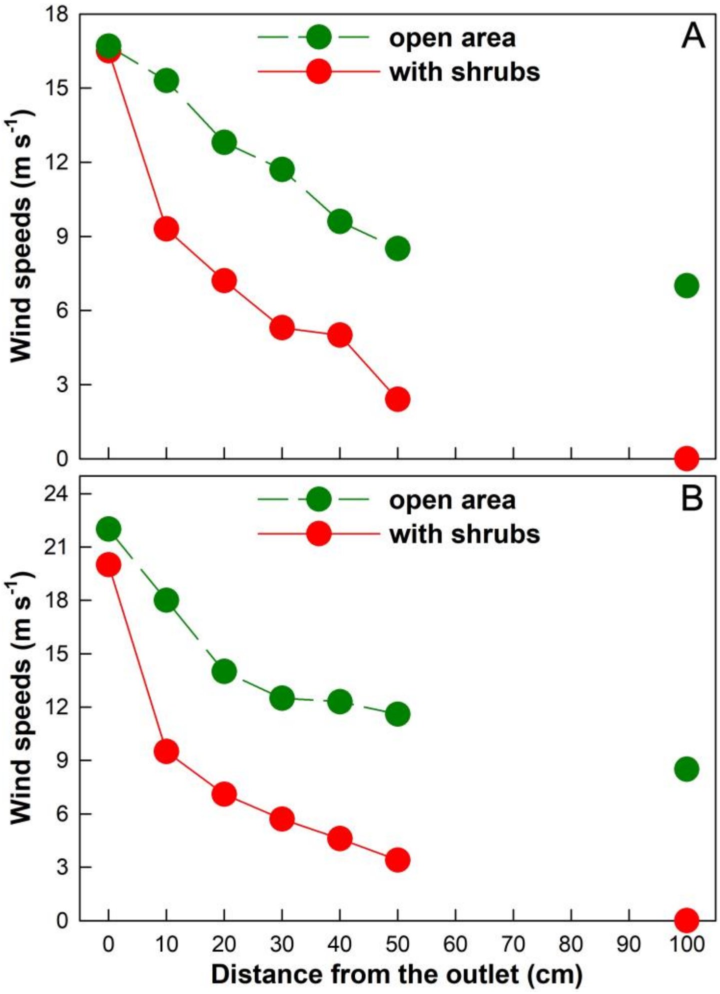

Figure S7. Random forests reveal the predictors for the spatial patterns of the changes in plant litter and soil dry mass losses under ecological restoration. The relative contributions of ecological restoration-induced changes (Δ) in vegetation height and cover, soil properties (soil moisture and bulk density (SM and SBD)), local climate conditions (mean annual temperature and precipitation (MAT and MAP) and maximum and minimum temperature (T_{max} and T_{min})), and geographical positions (latitude (Lat), longitude (Lon), and elevation (Ele)) to those changes in the losses of plant litter (*A*), soil dry mass (*B*), and total soil carbon (*C*) and nitrogen (*D*) under simulated wind erosion. The percentage increase in the mean squared error (%InMSE) was used to assess the importance of these predictors, with negative values representing the lack of importance. * $P < 0.05$ and ** $P < 0.01$.

The canopy height of each plot was determined by measuring and averaging the heights of at least five randomly selected plant individuals. Soil moisture at 10 cm depth in each plot was measured with a time-domain reflectometer (TDR 200, Spectrum Scientific Inc., USA). In addition, the bulk density of the top 10 cm soil was determined using a standard container with a volume of 100 cm^3 (5.5 cm in diameter and 5 cm in height). Rock fragments were removed from the bulk density samples using a 2-mm mesh. The gravimetrical water content of the bulk density samples was measured after desiccating for 48 hours at 105°C. Soil bulk density was calculated as the ratio of the soil dry mass to the container volume.

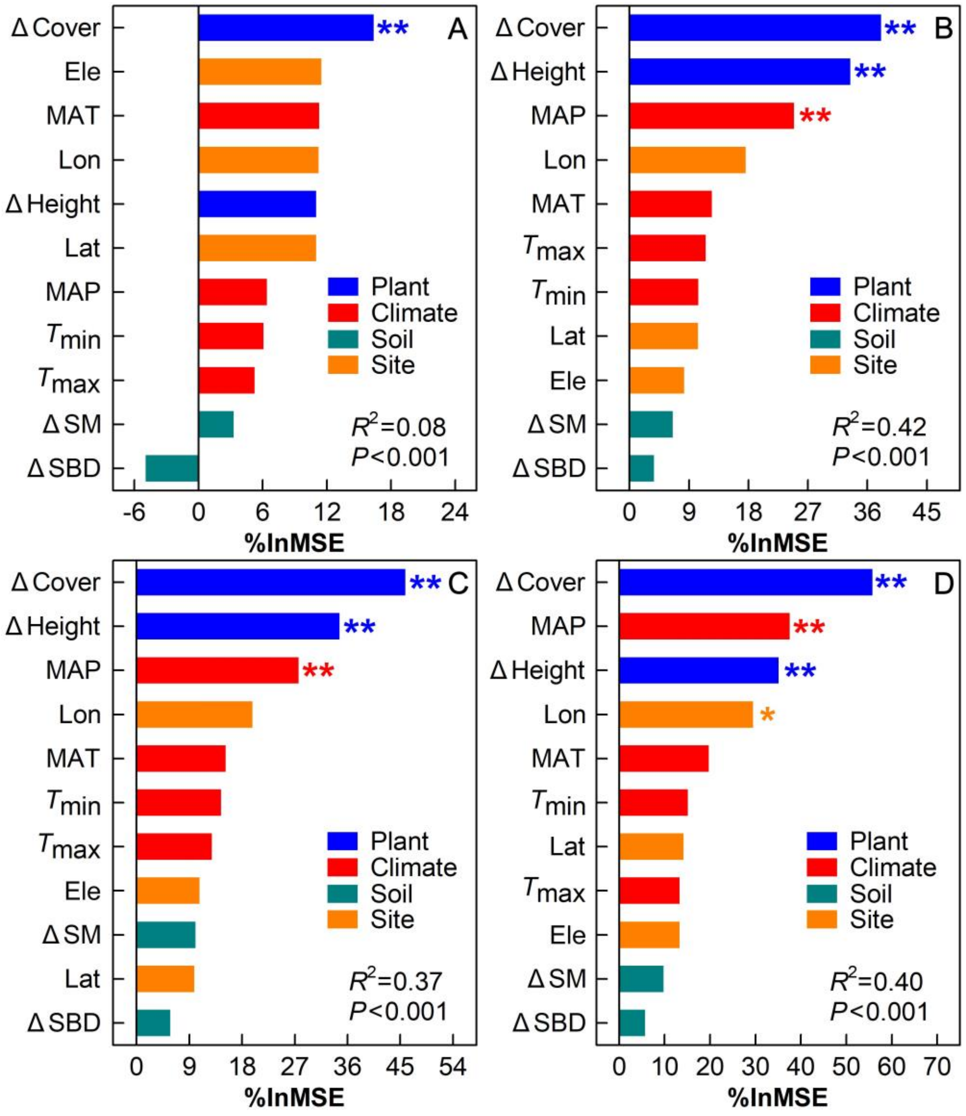

Figure S8. Decrease magnitudes of the losses of plant litter and soil dry mass in three ecosystems and seven ecological restoration types. Percentage changes (mean ± 95% CI, diamond with error bar) in wind erosion-induced losses of plant litter (*A*), soil dry mass (*B*), total soil carbon (C; *C*), and total soil nitrogen (N; *D*) in the three ecosystems (prior to ecological restoration) and under each of seven types of ecological restoration (T1−T7). Colored scatter plots represent percentage changes at each site. There is significant change if the 95% CI does not overlap zero. Significant between-group heterogeneity (Q_B; $P < 0.05$) indicates that the percentage changes differ among the three ecosystems or the seven restoration types. The red values in square brackets show sample size (number of sites with 25 m s^{-1} wind erosion). T1: grazed/degraded vs. ungrazed/undisturbed grasslands, T2: bare lands vs. disturbed/undisturbed grasslands/shrublands, T3: poorly developed vs. well-developed grasslands/shrublands, T4: deserts without vs. with desertification control, T5: croplands vs. natural grasslands, T6: croplands vs. old-field grasslands, and T7: bare soil vs. crop/no tillage with residue retention.

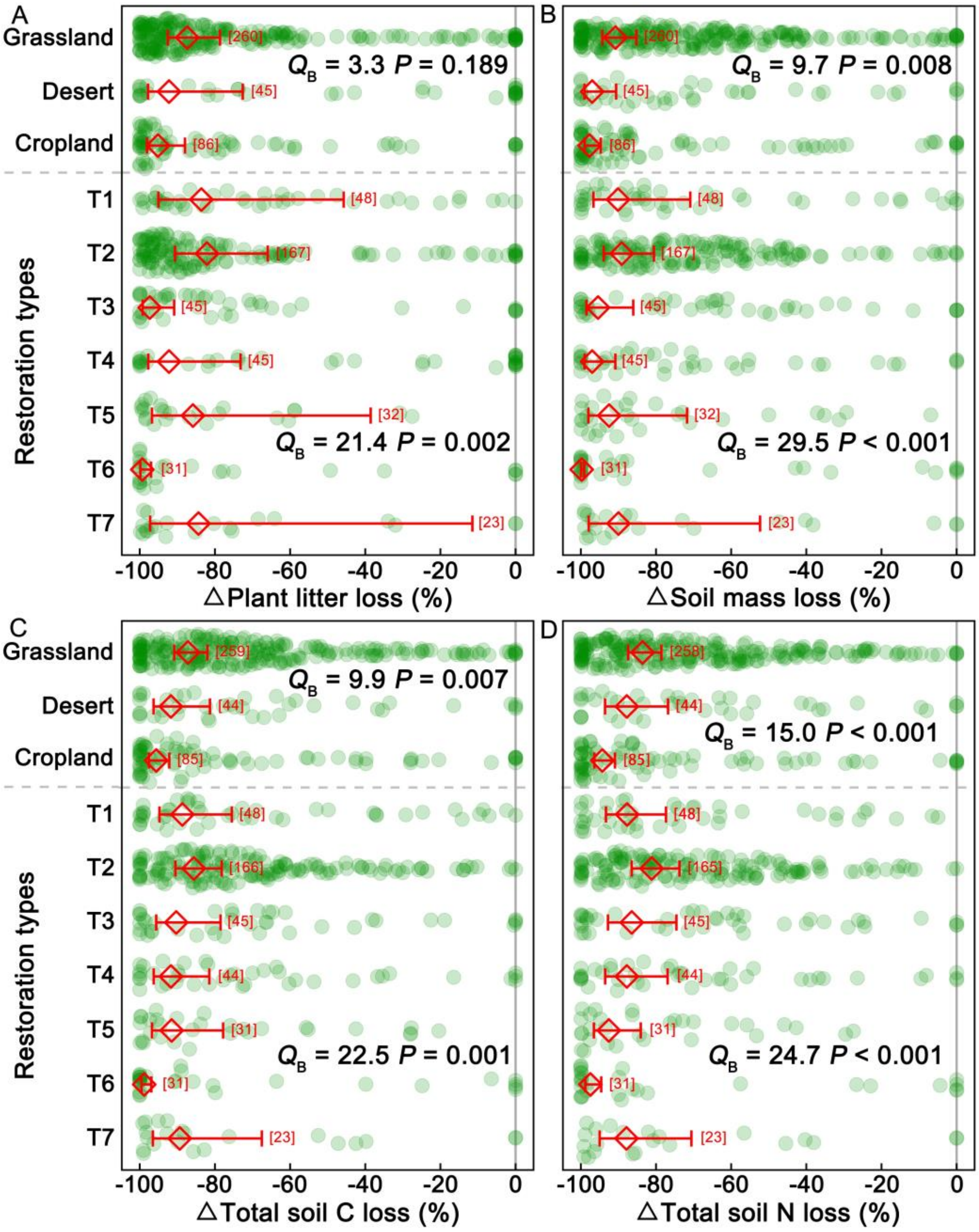

Figure S9. Changes in total soil carbon (C) and nitrogen (N) concentrations. Percentage changes (mean ± 95% CI, diamond with error bar) in total soil C (*A*) and N concentrations (*B*) under ecological restoration in different ecosystems (prior to ecological restoration). Colored scatter plots represent percentage changes at each site. There is significant change if the 95% CI does not overlap zero. Significant between-group heterogeneity (Q_B; $P < 0.05$) indicates that the percentage changes differ among the three ecosystems. The red values in square brackets show sample size (number of sites).

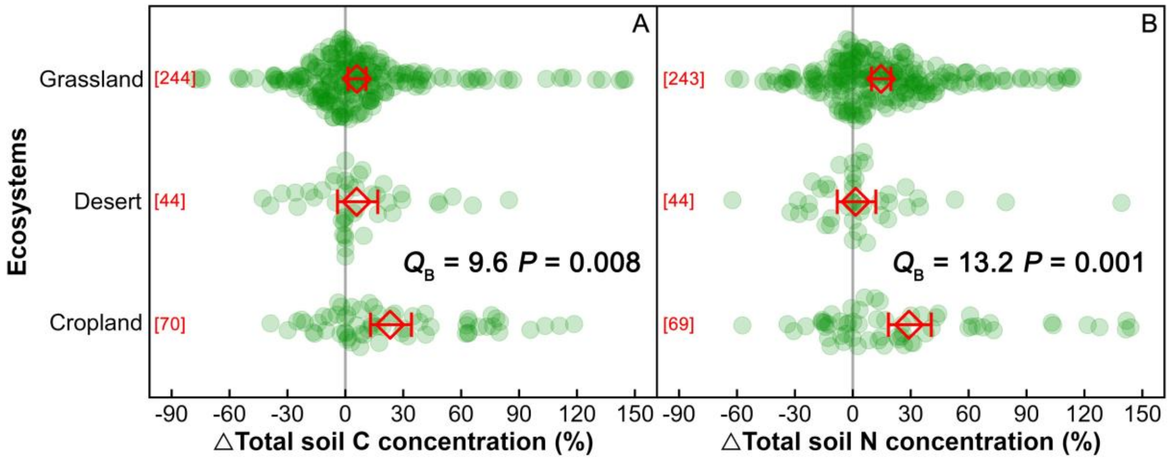

Figure S10. Random forests reveal the predictors for the spatial patterns of the changes in total soil carbon (C) concentration under ecological restoration. The relative contributions of the ecological restoration-induced changes (Δ) in vegetation cover and total soil C loss, soil properties (soil moisture and bulk density (SM and SBD)), local climate conditions (mean annual precipitation and temperature (MAP and MAT) and maximum and minimum temperature (T_{max} and T_{min})), and geographical positions (latitude (Lat), longitude (Lon), and elevation (Ele)) to the changes in total soil C concentration under ecological restoration (*A*). The percentage increase in the mean squared error (MSE) was used to assess the importance of these predictors, with negative values representing the lack of importance. * $P < 0.05$ and ** $P < 0.01$. Relationships of response ratios (RRs) of total soil C concentration under ecological restoration with MAP (*B*) and MAT (*C*) and T_{max} (*D*). Each data point represents the RRs of a single site.

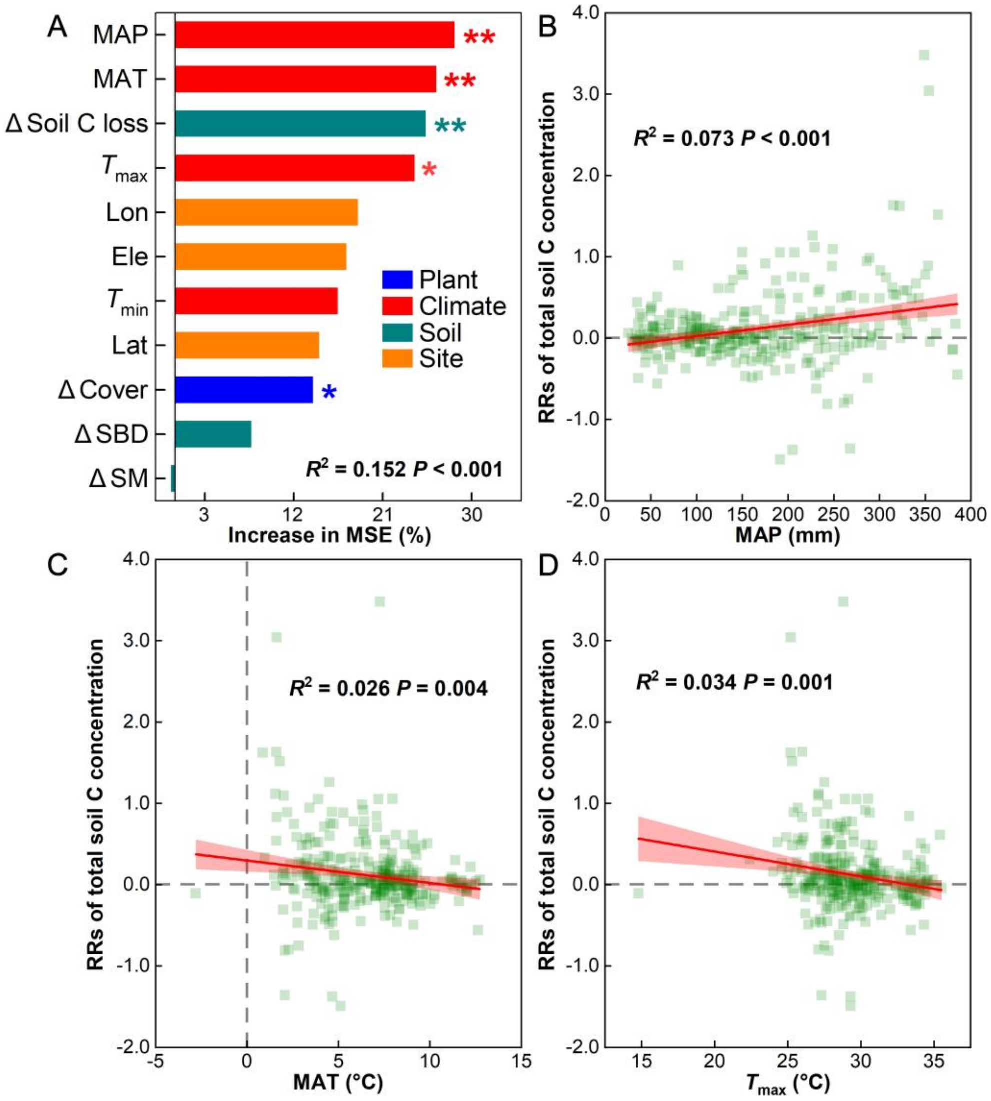

Figure S11. Total soil carbon (C) and nitrogen (N) losses caused by wind erosion and soil N availability. Annual losses of total soil C (*A*) and N (*B*) from the WEG and WE plots caused by the simualted wind erosion. Soil available N content under the four treatments averaged across five years from 2018 to 2022 (*C*). Different upper case letters above the bars represent significant differences among the four treatments ($P < 0.05$, multiple comparisons). Mean ± 1 SE, $n = 5$. WEG: wind erosion plus grazing, WE: wind erosion only, G: grazing only, and Ct: control.

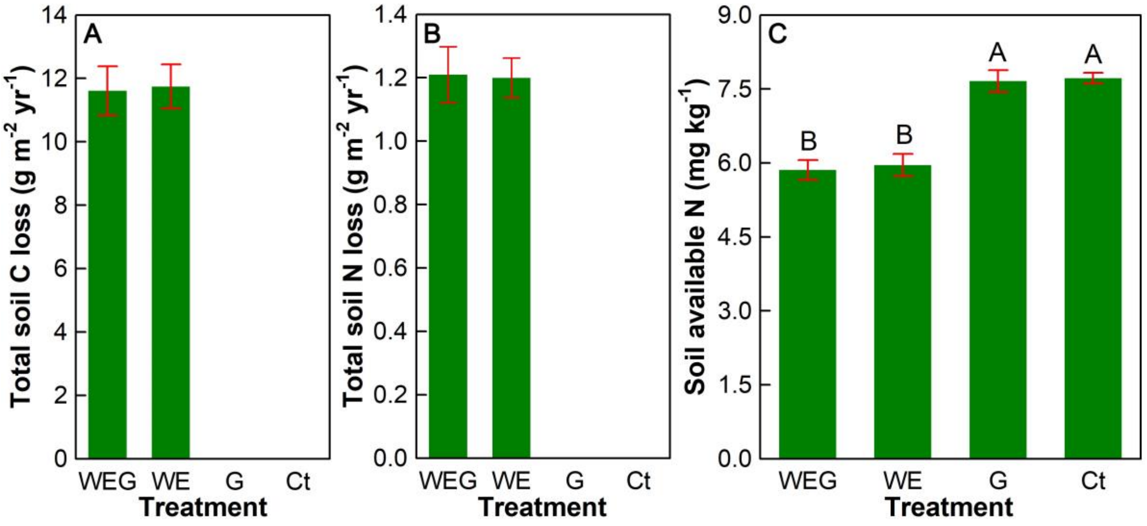

Figure S12. Changes in total soil carbon (C) concentration with restoration duration. Absolute (*A*) and relative changes (*B*) in total soil C concentration under the three restoration scenarios during the 13-year experimental period. WEG: wind erosion plus grazing, WE: wind erosion only, G: grazing only, Ct: control. Ct−WEG: difference between Ct and WEG represents restoration effect compared with the severe degradation with both wind erosion plus grazing; Ct−WE: difference between Ct and WE represents restoration effect compared with the moderate degradation with wind erosion only; Ct−G: difference between Ct and G represents rrestoration effect compared with the slight degradation with grazing only.

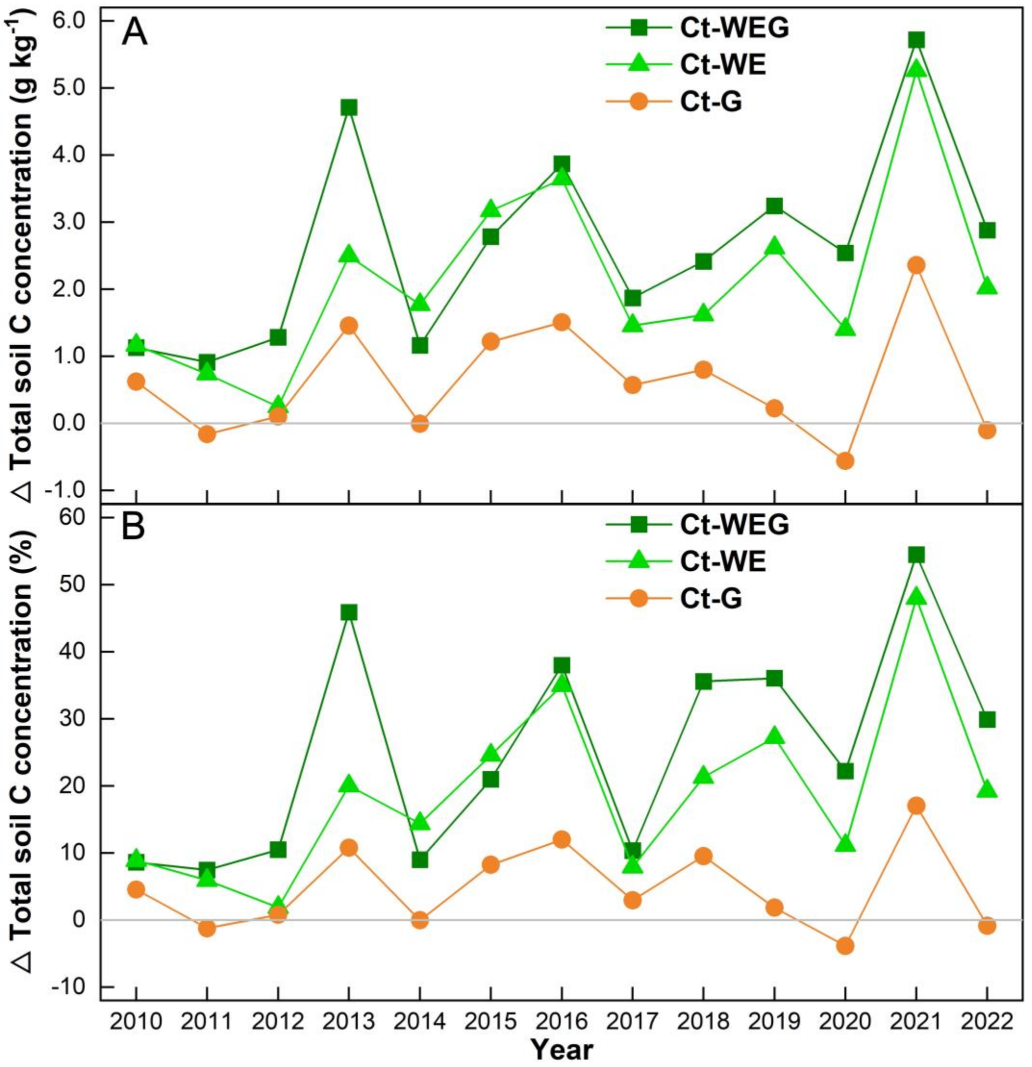

Figure S13. Changes in total soil nitrogen (N) concentration with restoration duration. Absolute (*A*) and relative changes (*B*) in total soil N concentration under the three restoration scenarios during the 13-year experimental period. WEG: wind erosion plus grazing, WE: wind erosion only, G: grazing only, and Ct: control. Ct−WEG: difference between Ct and WEG represents restoration effect compared with the severe degradation with wind erosion plus grazing, Ct−WE: difference between Ct and WE represents restoration effect compared with the moderate degradation with wind erosion only, Ct−G: difference between Ct and G represents restoration effect compared with the slight degradation with grazing only.

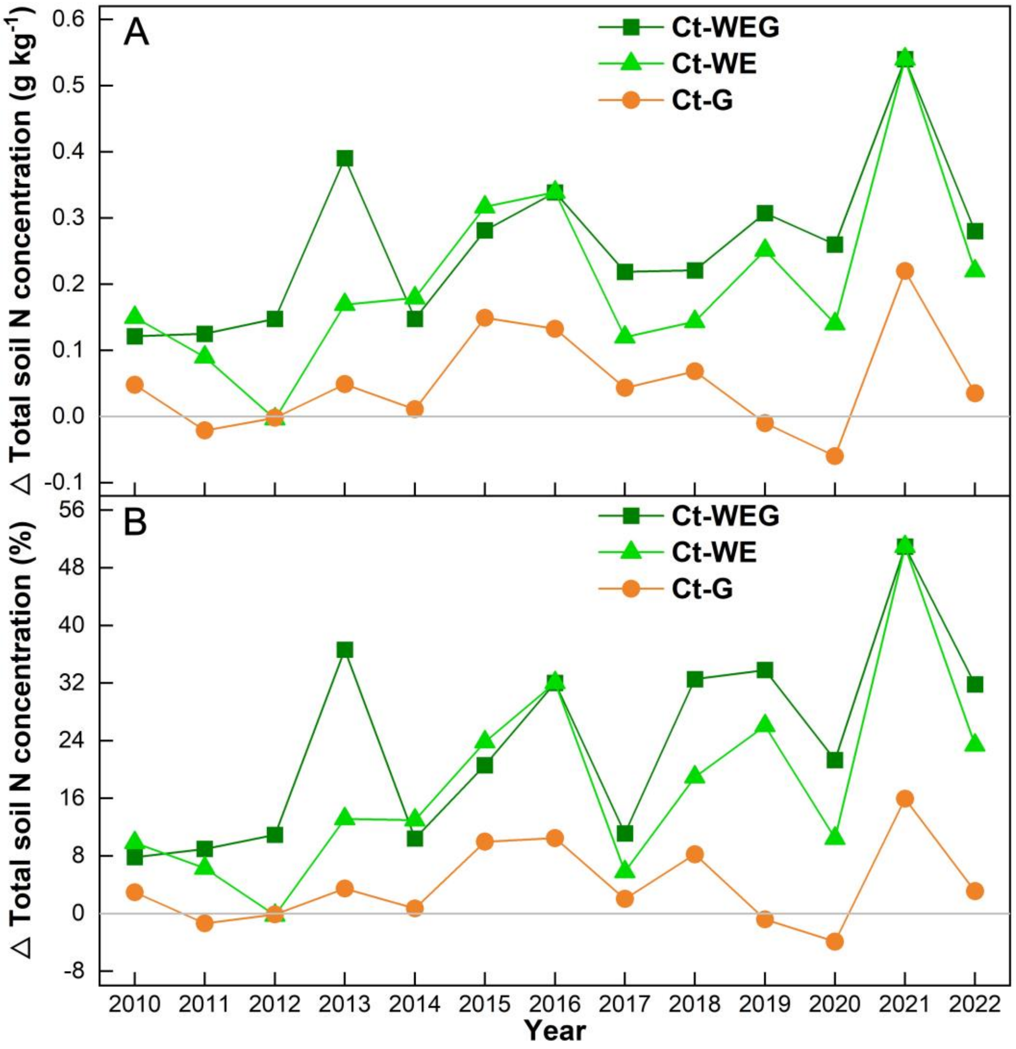

Figure S14. Random forests reveal the predictors of variations in plant cover and net primary productivity (NPP). The relative contributions of soil temperature (T), soil moisture (M), and total soil nitrogen (N) and soil inorganic nitrogen (SIN) concentrations to plant cover (*A*) and NPP (*B*) averaged over the 13 years from 2010 to 2022. The percentage increase in the mean squared error (MSE) was used to assess the importance of these predictors, with negative values representing the lack of importance. * $P < 0.05$ and ** $P < 0.01$.

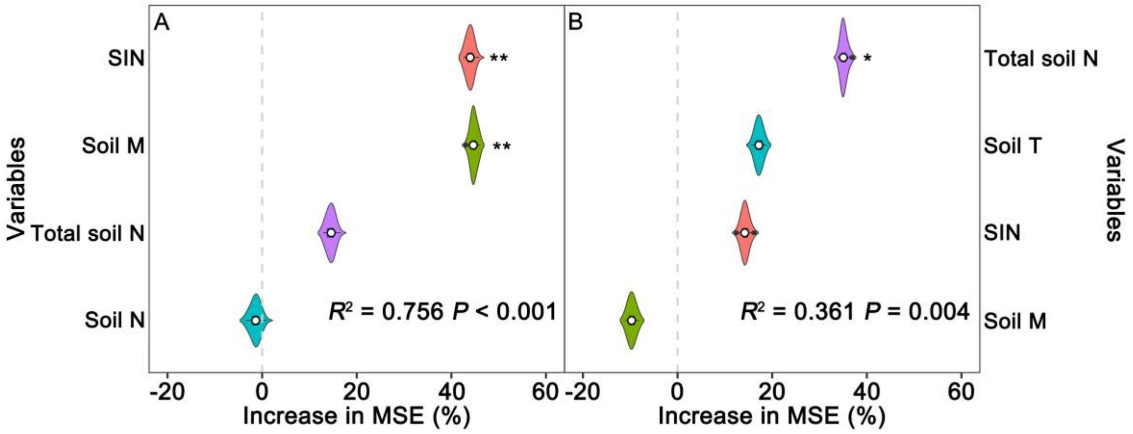

Figure S15. Changes in net primary productivity (NPP) with restoration duration. Absolute (*A*) and relative changes (*B*) in NPP under the three restoration scenarios during the 13-year experimental period. WEG: wind erosion plus grazing, WE: wind erosion only, G: grazing only, and Ct: control. Ct−WEG: difference between Ct and WEG represents restoration effect compared with the severe degradation with wind erosion plus grazing, Ct−WE: difference between Ct and WE represents restoration effect compared with the moderate degradation with wind erosion only, Ct−G: difference between Ct and G represents restoration effect compared with the slight degradation with grazing only.

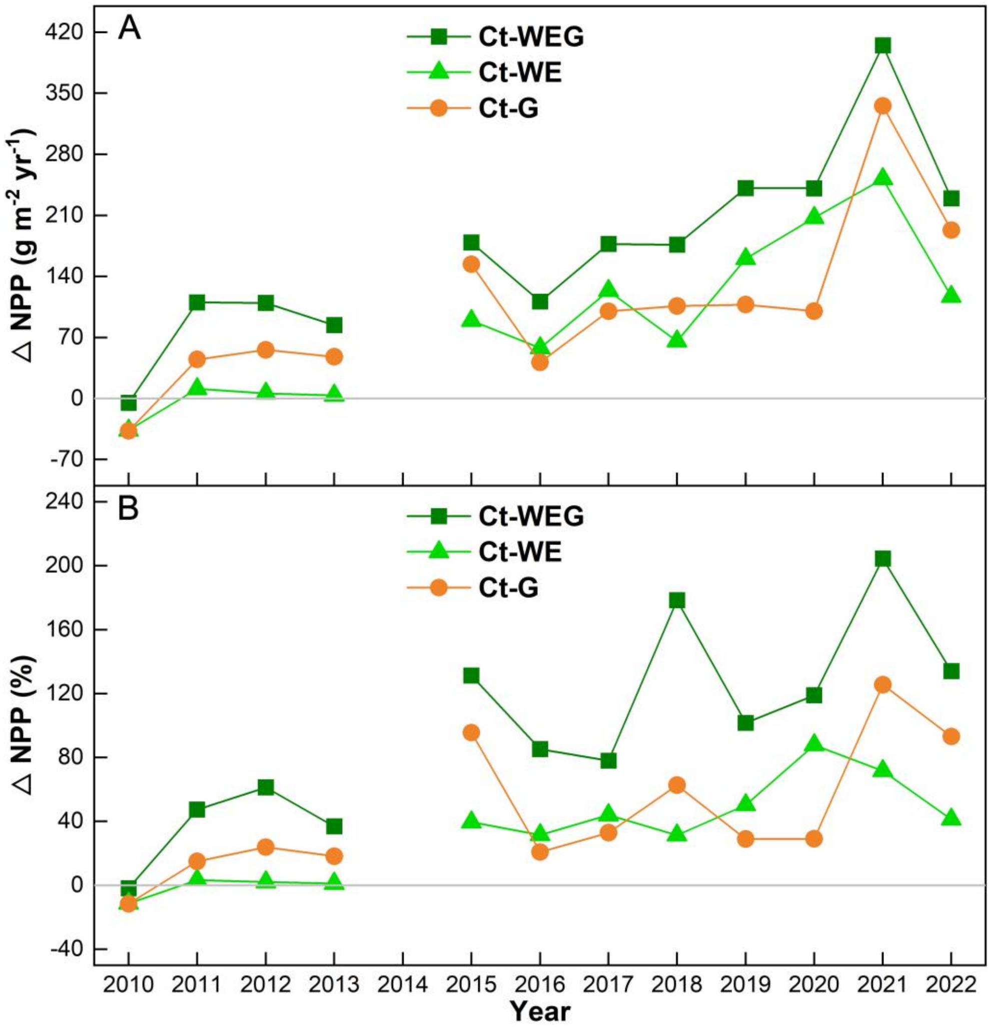

Figure S16. Responses of soil organic carbon to ecological restoration based on data from a previous regional survey conducted from 2009 to 2010. Soil organic carbon density in the upper 1 meter under degraded and restored conditions across six major grassland types of China. Numerical values above the bars represent the number of paired observations. Adapted from Song *et al.* (2018).

J. Song *et al.,* The carbon sequestration potential of China's grasslands. *Ecosphere* **9,** e02452 (2018).

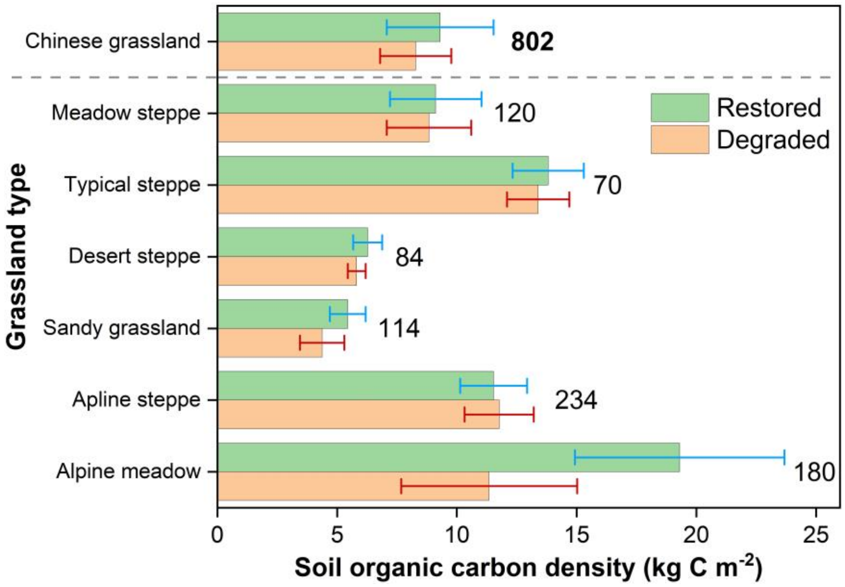

Figure S17. Total soil carbon (C) concentration and soil respiration rates under both wind erosion and dust deposition. Thirteen-year (2010−2022) means (mean ± 1 SE, diamond with error bar) of total soil carbon (C) concentration (*A*) and soil respiration (*B*) under the six treatments. Colored scatter plots represent values in each plot each year. *n* = 65 (5 replicates × 13 years) for each treatment. The gray line represents the mean value in the Ct plots. Ct: control, WE: wind erosion, DD: dust deposition, G: grazing, WEG: wind erosion plus grazing, DDG: dust deposition plus grazing.

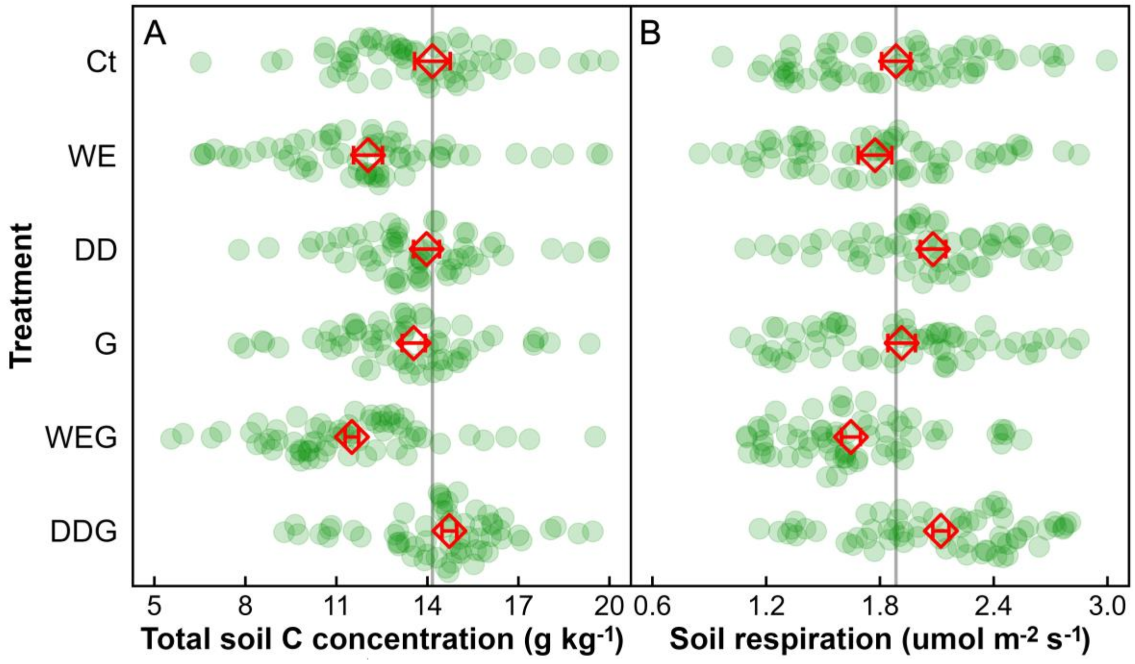

Figure S18. Grassland protection area during 2001−2021. The cumulative area of grassland fencing, the annual implementation area of artificial grass planting and grassland improvement, as well as the areas under different grazing practices, such as grazing prohibition and rotational grazing, in grasslands across North and Northwest China during the past two decades. Data sources: National Grassland Annual Monitoring Report (Grassland Supervision Center, Ministry of Agriculture and Rural Affairs of the People's Republic of China), National Forestry and Grassland Development Report, and Bulletin on the Greening of China's Land (National Forestry and Grassland Administration).

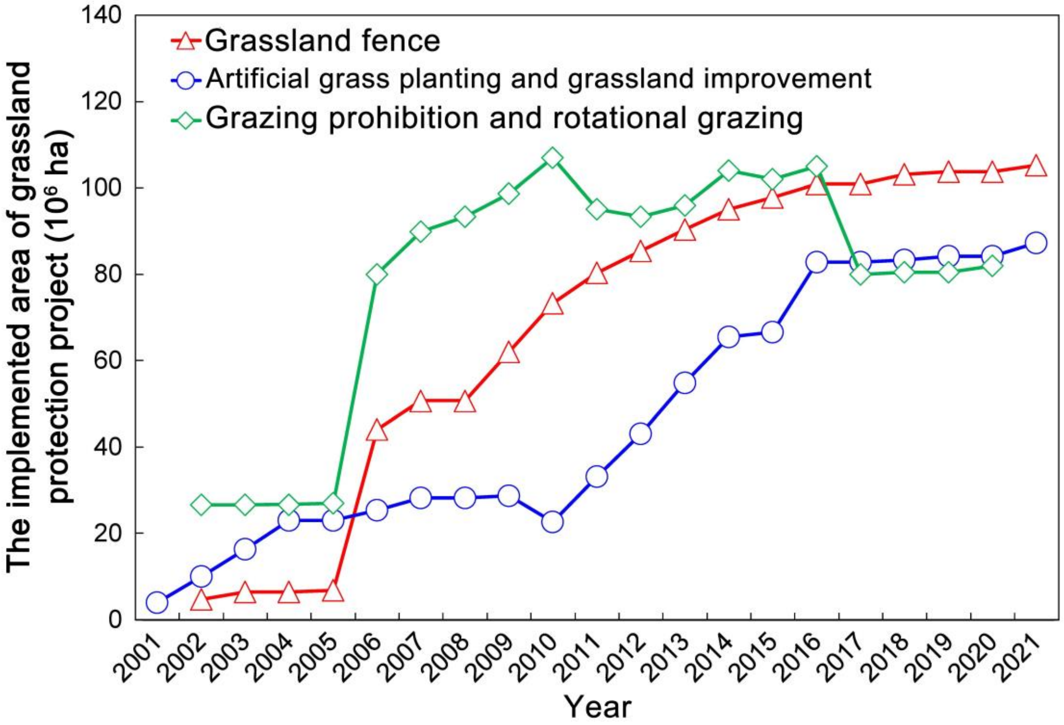

Figure S19. Proportion of different restoration types in the field survey. The proportion of each of the seven restoration types in the first (spring 2014; *A*), second (autumn 2014; *B*), third (spring 2015; *C*), and fourth (spring 2016; *D*) transect surveys in North and Northwest China. T1: grazed/degraded vs. ungrazed/undisturbed grasslands, T2: bare lands vs. disturbed/undisturbed grasslands/shrublands, T3: poorly developed vs. well-developed grasslands/shrublands, T4: deserts without vs. with desertification control, T5: croplands vs. natural grasslands, T6: croplands vs. old-field grasslands, and T7: bare soil vs. crop/no tillage with residue retention.

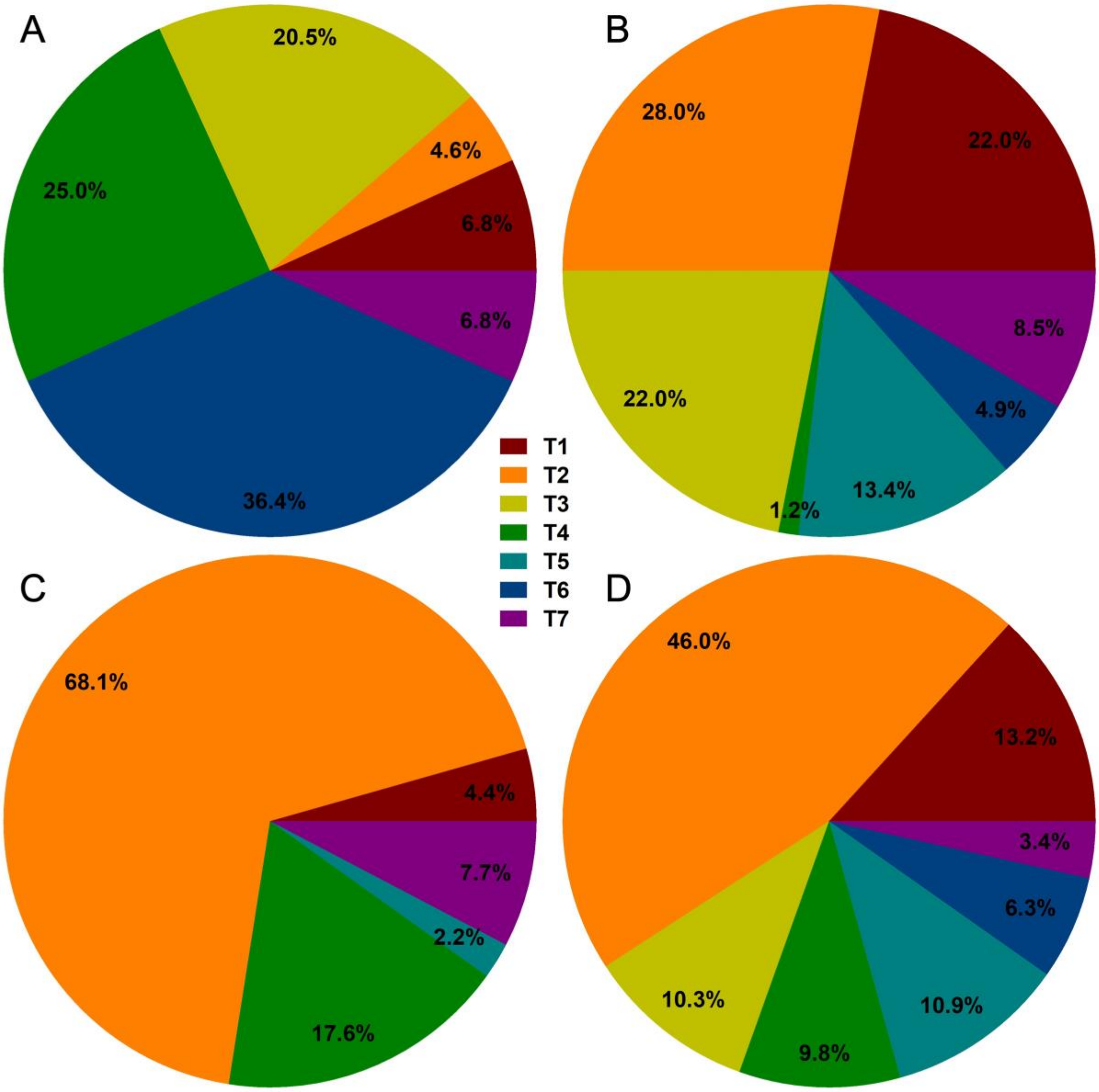

Figure S20. Design, tests, and member training of the modified pneumatic extinguisher. In March 2014, field testing and member training of the modified pneumatic extinguisher with adjustable wind speeds was conducted (*A*-*D*), along with wind speed recordings at various distances from the air outlet under different fuel feeding (FF) levels (low, medium, and high; *E*). The modified pneumatic extinguisher is patented by China National Intellectual Property Administration (*F*).

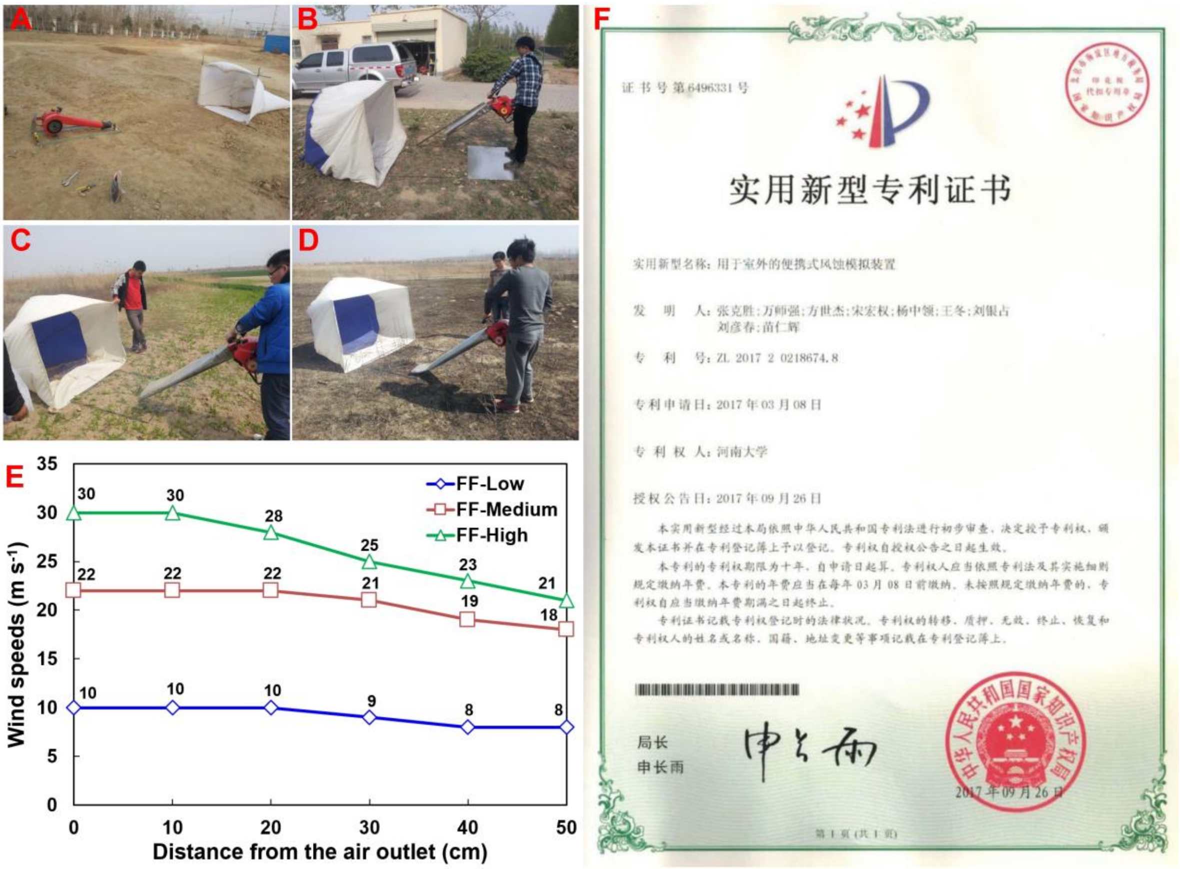

Figure S21. Responses of subsoil carbon concentrations to ecological restoration. Responses ratios (mean ± 95% CI, diamond with error bar) of total soil carbon concentration at depths of 10-20 cm (*A*), 20-40 cm (*B*), and 40-60 cm (*C*) in response to ecological restoration averaged across all (Overall) as well as in three ecosystems (prior to ecological restoration) and each of seven types of ecological restoration (T1−T7). Colored scatter plots represent percentage changes at each site. There is significant change if the 95% CI does not overlap zero. The red values along the y-axis denote sample sizes (number of sites). Ecological restoration types include: T1: grazed/degraded vs. ungrazed/undisturbed grasslands, T2: bare lands vs. disturbed/undisturbed grasslands/shrublands, T3: poorly developed vs. well-developed grasslands/shrublands, T4: deserts without vs. with desertification control, T5: croplands vs. natural grasslands, T6: croplands vs. old-field grasslands, and T7: bare soil vs. crop/no tillage with residue retention.

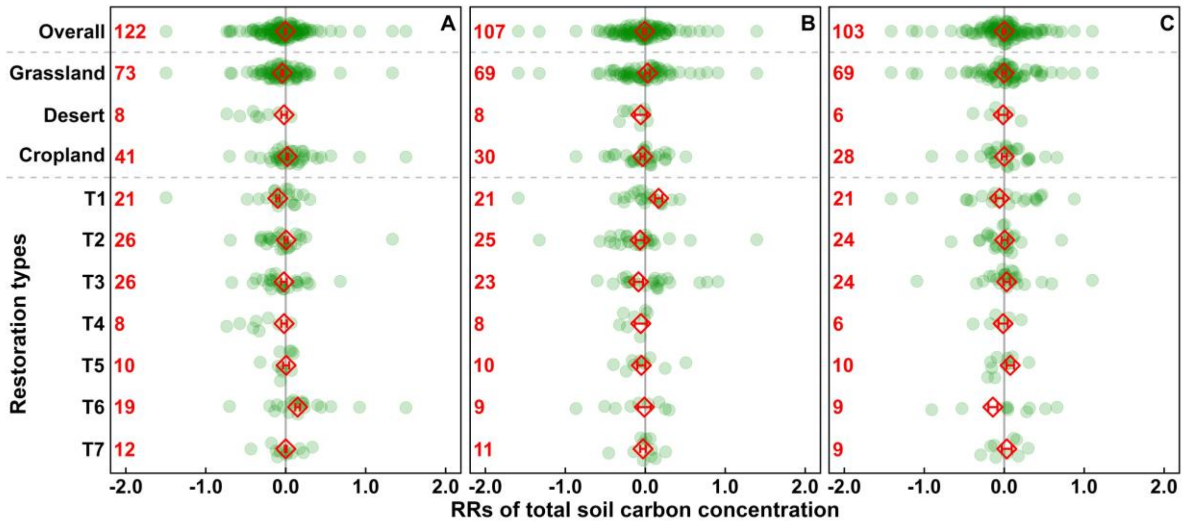

Table S1. Numbers of the sampling sites and plots in the four transect surveys in North and Northwest China.

				All the five WSs		WS of 12 m s^{-1}		WS of 16 m s^{-1}		WS of 21 m s^{-1}		WS of 25 m s^{-1}		WS of 30 m s^{-1}	
	**Periods of time**	**Distance (km)**	**Province**	**NSa**	**NSps**	**NSa**	**NSps**	**NSa**	**NSps**	**NSa**	**NSps**	**NSa**	**NSps**	**NSa**	**NSps**
**S1**	Apr-May 2014	>14000	Inner Mongolia	101(1902)	44(1116)	94(441)	44(264)	94(441)	44(264)	94(441)	44(264)	95(444)	44(264)	16(102)	10(60)
**S2**	Sep-Oct 2014	>6000	Inner Mongolia	82(492)	82(492)	0	0	0	0	0	0	82(492)	82(492)	0	0
**S3**	Apr-Jun 2015	>20000	Gansu	2(12)	2(12)	0	0	0	0	0	0	2(12)	2(12)	0	0
			Xinjiang	126(670)	88(528)	1(4)	0	1(4)	0	1(4)	0	126(670)	88(528)	0	0
			Qinghai	13(57)	1(6)	0	0	0	0	0	0	13(57)	1(6)	0	0
**S4**	Apr-Jun 2016	>20000	Inner Mongolia	135(801)	117(702)	0	0	0	0	0	0	135(801)	117(702)	0	0
			Ningxia	5(30)	5(30)	0	0	0	0	0	0	5(30)	5(30)	0	0
			Gansu	53(315)	52(312)	0	0	0	0	0	0	53(315)	52(312)	0	0
**S1−S4**	2014-2016	>60000	Inner Mongolia	318(3195)	243(2310)	94(441)	44(264)	94(441)	44(264)	94(441)	44(264)	312(1737)	243(1458)	16(102)	10(60)
			Ningxia	5(30)	5(30)	0	0	0	0	0	0	5(30)	5(30)	0	0
			Gansu	55(327)	54(324)	0	0	0	0	0	0	55(327)	54(324)	0	0
			Xinjiang	126(670)	88(528)	1(4)	0	1(4)	0	1(4)	0	126(670)	88(528)	0	0
			Qinghai	13(57)	1(6)	0	0	0	0	0	0	13(57)	1(6)	0	0
			Overall	517(4279)	391(3198)	95(445)	44(264)	95(445)	44(264)	95(445)	44(264)	511(2821)	391(2346)	16(102)	10(60)

Major abbreviations: S1, S2, S3, and S4 represent the first, second, third, and fourth transect surveys, respectively. NSa refers to number of all sampling sites in each and all of the five wind speeds (WS) including 12, 16, 21, 25, and 30 m s^{-1}. NSps represents number of the sites with pairwise sampling in each and all of the five wind speeds. Numbers in the parentheses are the number of $1\times1m^2$ plots.

Table S2. Wind erosion dominating the soil erosion in our study area. Statistics of wind and water erosion: Area and contribution to soil loss in our study area. (Data source: Spatial Distribution of Soil Erosion in China; Figure S1*C*).

**Erosion type**	**Intensity**	**Area (km²)**	**Erosion intensity (t/km²·a)**	**Total erosion (10^6 t/a)**	**Proportion**
**Water**					
	Slight	472,341	<200, 500, 1000--500	236.2	
	Light	250,579	200, 500, 1000~2500--1500	375.9	
	Moderate	132,811	2500~5000--3750	498.0	
	Strong	44,276	5000~8000--6500	287.8	
	Severe	31,361	8000~15000--11500	360.7	
	Intense	14,358	>15000--15000	215.4	
	**total**	**945,726**		1,974.0	10.9%
**Wind**					
	Slight	269,511	<200, 500, 1000--500	134.8	
	Light	316,459	200, 500, 1000~2500--1500	474.7	
	Moderate	342,839	2500~5000--3750	1285.6	
	Strong	364,150	5000~8000--6500	2,367.0	
	Severe	376,683	8000~15000--11500	4,331.9	
	Intense	503,679	>15000--15000	7,555.2	
	**total**	**217,3321**		16,149.2	89.1%
			**total**	**18,123.2**	

Table S3. Classification of seven types of ecological restoration. The identification of the sampling pairs without and with ecological restoration measures in the seven ecological restoration types, including T1: grazed/degraded vs. ungrazed/undisturbed grasslands, T2: bare lands vs. disturbed/undisturbed grasslands/shrublands, T3: poorly developed vs. well-developed grasslands/shrublands, T4: deserts without vs. with desertification control, T5: croplands vs. natural grasslands, T6: croplands vs. old-field grasslands, and T7: bare soil vs. crop/no tillage with residue retention.

	**Sampling pairs**	
**Types**	**Degraded/disturbed**	**Restored/undisturbed**
**T1**	Grazed steppes	Fenced steppes
	Overgrazed steppes	Lightly/Moderately grazed steppes
	Moderately grazed steppes	Lightly grazed steppes
	Mowed grasslands/semi-shrublands	Unmowed grasslands/semi-shrublands
	Severely degraded grasslands	Moderately degraded grasslands
	Steppes with rodent damage	Steppes with rodent control
	Burned steppes	Unburned steppes
**T2**	Bare lands	Desert/typical steppes
	Bare lands	Shrublands/Tree seedlings (e.g., pines)
**T3**	Desert/typical/meadow steppes/Old-fields	Dense shrublands
	Old-fields	Typical steppes
	Desert/typical steppes	Meadow steppes
**T4**	Deserts	Straw checkerboards/Dense shrublands
	Gobi/Degraded shrublands	Desert steppes/Dense shrublands
**T5**	Tillage soil without crops	Grazed/desert/typical steppes/Pasture lands
	Tillage soil without crops	Shrublands/Tree seedlings
	Wheat/corn fields (Single crop field)	Desert/typical steppes
**T6**	Tillage soil without crops	Old-fields
	Alfalfa/wheat/corn fields (Single crop field)	Old-fields
**T7**	Low-cover farmlands (Single crop field)	High-cover farmlands (Single crop field)
	Burned no-tillage farmland	Unburned with crop stubble
	Fallow fields	Corn fields (Single crop field)
	Tillage soil without crops	Wheat/corn/cotton fields (Single crop field) or Fallow fields
	Tillage soil without crops	No-tillage farmlands with crop stubble

Legend for the supplementary vedio: Video on a sandy storm met by the team at 15:56 pm (Beijing Time) May 20, 2016 during the fourth transect survey.
